# Single-cell and bulk RNA sequencing reveals Anoikis related genes to guide prognosis and immunotherapy in osteosarcoma

**DOI:** 10.1038/s41598-023-47367-3

**Published:** 2023-11-18

**Authors:** Cheng Zhong, Dongliang Yang, Liping Zhong, Weixing Xie, Guodong Sun, Daxiang Jin, Yuming Li

**Affiliations:** 1 Department of Orthopedics, The First Clinical Medical College of Guangzhou University of Chinese Medicine, Guangzhou, 515000 China; 2 Department of Orthopedics, Jiangmen Hospital of Traditional Chinese Medicine Affiliated to Jinan University, Jiangmen, 529000 China; 3 Department of Orthopedics, Tai Shan Hospital of Traditional Chinese Medicine Affiliated to Guangzhou University of Chinese Medicine, Jiangmen, 529000 China; 4 Department of Cardiothoracic Surgery, Jiangmen Hospital of Traditional Chinese Medicine Affiliated to Jinan University, Jiangmen, 529000 China; 5 Department of Orthopedics, The First Affiliated Hospital of Jinan University, Guangzhou, 510630 China

**Keywords:** Cancer, Biomarkers, Cancer

## Abstract

Anoikis resistance, a notable factor in osteosarcoma, plays a significant role in tumor invasion and metastasis. This study seeks to identify a distinct gene signature that is specifically associated with the anoikis subcluster in osteosarcoma. Clinical, single-cell, and transcriptional data from TARGET and GEO datasets were used to develop a gene signature for osteosarcoma based on the anoikis subcluster. Univariate Cox and LASSO regression analyses were employed. The signature's predictive value was evaluated using time-dependent ROC and Kaplan–Meier analyses. Functional enrichment analyses and drug sensitivity analyses were conducted. Validation of three modular genes was performed using RT-qPCR and Western blotting. Signature (ZNF583, CGNL1, CXCL13) was developed to predict overall survival in osteosarcoma patients, targeting the anoikis subcluster. The signature demonstrated good performance in external validation. Stratification based on the signature revealed significantly different prognoses. The signature was an independent prognostic factor. The low-risk group showed enhanced immune cell infiltration and improved immune function. Drug sensitivity analysis indicated efficacy of chemotherapy agents. Prognostic nomograms incorporating the signature provided greater predictive accuracy and clinical utility. Signatures related to the anoikis subcluster play a significant role in osteosarcoma progression. Incorporating these findings into clinical decision-making can improve osteosarcoma treatment and patient outcomes.

## Introduction

Osteosarcoma (OS) is a highly aggressive primary neoplasm with a predilection for the pediatric and young adult population, demonstrating a high fatality rate^1,2^. OS affects 2–4 million individuals globally annually, with the highest incidence observed in the age group of 15–18 years^3^. Typically, OS occurs in the metaphysis of the long diaphysis and exhibits characteristics of metastasis^1,4^. According to statistics, the survival rate of OS with surgery alone is 15–17%^5^. In addition to surgery and radiotherapy, the development of adjuvant chemotherapy has increased the survival rate of OS to 60%, then reaching the plateau^6^. However, with the occurrence of drug resistance, the survival rate of OS decreases statistically, leading to poor prognosis^7^. Despite the utilization of standard therapeutic interventions, the 5-year survival rate for recurrent metastatic osteosarcoma remains below 20%^8^. Identifying novel therapeutic targets and prognostic markers is crucial for improving patient outcomes in OS^9^.

Apoptosis, triggered by diverse factors and pathways, encompasses the loss of cellular adhesion to the extracellular matrix (ECM) and aberrant adhesion as crucial elements^10^. Anoikis resistance is thought to initiate tumor invasion and metastasis. Therefore, in-depth studies of anoikis resistance will yield crucial insights into cancer metastasis^11^. Anoikis resistance has been observed to develop during tumor progression, anoikis-dependent metastasis, as well as events such as autophagy and metabolic changes, indicating its importance in tumor biology^12,13^. Although the association of anoikis with tumor progression and metastasis is well-known, the role of anoikis-related genes (ANRG) in osteosarcomas has been poorly studied.

Two distinct subtypes of anoikis were identified in the immune microenvironment in our study of ANRGs in osteosarcoma. We developed and validated a new signature consisting of modular genes related to anoikis subclusters for precise prognostication of osteosarcoma. Our study also investigated the relationship between osteosarcoma risk-scoring signatures and immune cell infiltration, immune checkpoint modulation, and pharmacological response. Validation of our model has demonstrated its accuracy in predicting osteosarcoma outcomes. Our findings shed light on how regulatory profiles related to anoikis contribute to osteosarcoma-hostile behavior, providing a novel perspective on osteosarcoma treatment protocols. The identified anoikis subcluster-related modular genes were also analyzed in vitro and in pancancer studies.

## Materials and methods

### Data sources and processing

The analysis involved 85 osteosarcoma mRNA expression profiles and their corresponding clinical characteristics obtained from the TARGET database (https://ocg.cancer.gov/programs/target). Furthermore, mRNA expression profiles and relevant clinical data of 42 osteosarcoma patients were analyzed using the GEO database (accession number GSE39058). Exclusion criteria were applied to samples that lacked clinical follow-up information, had unknown survival time less than 30 days, or had no survival status. RNA sequencing data of gene expression (measured in FPKM values) and clinical information from the TARGET dataset were directly accessed through the UCSC XENA dataset (https://xena.ucsc.edu/). The normalized matrix files for microarray data from the Illumina platform were obtained directly from the Gene Expression Omnibus (GEO) dataset under accession number GSE39058 (http://www.ncbi.nlm.nih.gov/geo/). The validation dataset used was GSE21257 retrieved from the GEO database, while GSE42352 was used for conducting difference analysis. Previously, the TPM (transcripts per kilobase million) values were stated to be commensurate with the microarray data^14,15^. The FPKM data was transformed into TPM data. To conduct further analysis, any batch effects present between these cohorts were eliminated using the "sva" R package.

In the GSE152048 dataset, single-cell RNA sequencing data from 11 osteosarcoma tissues were obtained using the 10X Genomics platform. To comprehensively process the single-cell RNA sequencing (scRNA-seq) data, a series of systematic steps were executed. Initially, the scRNA-seq data was preprocessed using the "Seurat" package. Subsequently, a combination of procedures, including the PercentageFeatureSet function, was employed to evaluate the proportion of mitochondrial genes. Additionally, correlation analysis was performed to examine the association between sequencing depth, mitochondrial gene sequences, and total intracellular sequences.

In order to ensure robust analysis, genes were filtered to ensure a minimum expression level in at least 5 cells. Moreover, a stringent selection process was implemented to retain cells that met specific criteria, including a gene expression count ranging from more than 300 to less than 5000, mitochondrial content below 10%, and a minimum UMI count of 1000 per cell. Once the data filtering steps were completed, normalization of the scRNA-seq data was performed using the LogNormalize method. This normalization step aimed to facilitate accurate downstream analysis and interpretation. "Diploid" refers to "cells with a regular chromosomal count", while "aneuploid" indicates "cells with irregular chromosomal numbers, often found in tumors". The term "not defined" relates to "cells with an undetermined chromosomal status"^16^.

### Detection of Anoikis-related genes and Identification of the Anoikis-related subtypes

Genes relevant to Anoikis were identified by searching the GeneCards (https://www.genecards.org/) and Harmonizome databases (https://maayanlab.cloud/Harmonizome/) using the keyword "Anoikis". Genes associated with Anoikis were filtered based on a relevance score threshold of 0.4. Subsequently, an unsupervised clustering analysis was performed using the R package consensusClusterPlus, utilizing the expression levels of these Anoikis-related genes (ANRGs). Resampled samples were randomly selected at an 80% proportion using the R package consensusClusterPlus, with a maximum of nine categories evaluated. The clustering distance was determined using the "Euclidean" method. The optimal number of clusters (K) was determined through cluster division analysis, and it was determined that K = 2 represented the optimal number of clusters. Subsequently, a survival curve was generated using the "survival" package in R, considering gene expression and clinical characteristics of the patient clusters. Furthermore, a heat map was created using the "pheatmap" package in R to visualize the results. Additionally, a principal component analysis (PCA) was performed to validate the observed gene expression patterns across the various clusters.

### Identification and consensus clustering of differentially expressed genes (DEGs) for the determination of Anoikis-related subclusters

An intergroup difference analysis was performed using the "limma" package, filtering genes based on fold change (FC) thresholds greater than 1 and adjusted p-value thresholds < 0.01. The three gene clusters were analyzed using a univariate Cox model with a significance threshold of 0.05 to identify survival-related differentially expressed genes. Gene clustering was conducted using the "Consensus Cluster Plus" R package. Additionally, survival analysis for various genotypes was performed using the R package "survival".

### Function enrichment analysis

In this study, the Gene Set Enrichment Analysis (GSEA) function from the ClusterProfiler R package was employed on the "c2.cp.kegg.v7.4.symbols.gmt" gene set sourced from the MSigDB database. Hallmark gene sets were examined for two subgroups, and the top five sets with a p-value below 0.05 were reported. Gene set variation analysis was conducted to identify functional pathway disparities between the subtypes. To perform gene ontology (GO) and Kyoto Encyclopedia of Genes and Genomes (KEGG) analysis on the differentially expressed genes (DEGs), the "clusterProfiler" R package was utilized, with a threshold of q-value less than 0.05 indicating statistical significance.

### Construction and validation of the risk-scoring model

The differentially expressed genes (DEGs) with prognostic potential were selected by establishing an univariate Cox model, and were subsequently incorporated into a Cox regression model using LASSO regularization. To prevent overfitting, we used the "glmnet" R package with LASSO regression to select genes with prognostic coefficients determined by lambda values (λ). Risk scores were calculated for each patient using the formula below, where Coefi represents the coefficients and xi represents the level of gene expression in each individual.

$$Risk score ={\sum }_{i=1}^{n}Coefi \times xi$$

The patients were classified into high- or low-risk groups based on the median value of the calculated riskscore. The predictive performance and discriminatory capability of the riskscore model were evaluated using Kaplan–Meier survival analysis and time-dependent receiver operating characteristic curves (ROCs) through the implementation of the "survival" and "timeROC" R packages. Both the training and validation cohorts were stratified into high- and low-risk groups, employing the median value of the patients' risk score. Furthermore, univariate and multivariate Cox regression analyses were conducted to determine the impact of the riskscore on the prognosis of osteosarcoma patients.

### Immune analysis of the Anoikis subcluster-related prognostic signature and response to drug sensitivity

The study employed the single-sample gene set enrichment analysis (ssGSEA) method to evaluate the differences in immune cell infiltration between high and low-risk populations. Additionally, the expression levels of immune checkpoints were comparatively analyzed to assess the potential of the riskscore as a predictor of response to immunotherapy. Furthermore, the oncoPredict R package was utilized to examine drug sensitivity across various patient cohorts and identify divergences in the efficacy of targeted treatments.

### Predictive nomogram development and validation

Univariate and multivariate Cox regression models were utilized to assess the predictive efficacy of the risk score in conjunction with clinical characteristics, such as age and gender. The Cox regression analyses identified independent prognostic factors with p-values below 0.05. An interactive nomogram was developed using the “regplot” package in R to predict overall survival at one, three, and five years. Calibration curves were constructed to evaluate the accuracy of the nomogram predictions. Kaplan–Meier survival analysis and time-dependent receiver operating characteristic (ROC) curves were employed to evaluate the significance and discriminative ability of survival outcomes at 1-, 3-, and 5-year intervals. The nomogram was further validated using decision curve analysis (DCA) curves.

### Cell culture

Human fetal osteoblasts (hFOB1.19) and U-2 osteosarcomas (U2OS) cell lines were acquired from the American Type Culture Collection (ATCC) and cultured in DMEM media (Gibco, USA) supplemented with 10% fetal bovine serum and 100 μg/mL of 1% penicillin–streptomycin. Cultures were maintained in a humidified atmosphere with 5% CO2 at 37 °C.

### RNA isolation and quantitative reverse transcription-polymerase chain reaction (qRT-PCR) analysis

This study verified the results using qRT-PCR after RNA isolation from hFOB1.19 and U2OS cells. RNAiso Plus (Trizol) reagent was used to extract total RNA, which was then quantified using a NanoDrop 2000 spectrometer for purity and concentration determination. The TSK301 Reverse Transcription System Kit was used to conduct RT reactions and quantitative real-time PCR following the manufacturer's instructions. SYBR Green RT-qPCR Master mix from Tsingke Biotechnology Co., Ltd. (Beijing, China) was used for qRT-PCR analysis, and primers were designed and synthesized by Suzhou GENEWIZ Bioengineering Co. Amplification was performed using 40 cycles of denaturation at 95 °C for 10 s, annealing at 60 °C for 30 s, and elongation at 60 °C for 30 s. The expression levels of the target genes were normalized using GAPDH as an internal reference, and the 2-ΔΔCT method was used for data analysis. Human U2OS and hFOB1.19 cell lines were cultured using DMEM media (Gibco, USA) enriched with 10% fetal bovine serum and 100 μg/mL of 1% penicillin–streptomycin under a humidified atmosphere with 5% CO_2_ at 37 °C following standard protocols. Cells in the logarithmic growth stage were used for subsequent studies when the confluence rate reached about 80%. Following is a list of primer sequences for each signature gene:

CGNL1: ACCAGACCTTGCCGTTCATTAT (sense primer).

CGNL1: TGACGTGGGAGTTGTGGATG (antisense primer).

CXCL13: GCTTGAGGTGTAGATGTGTCC (sense primer).

CXCL13: CCCACGGGGCAAGATTTGAA (antisense primer).

ZNF583: GCAAAACAGGGAAGAGTGTTCAT (sense primer).

ZNF583: TAAGCCAACTCCAAGCCTGA (antisense primer).

GADPH: GAACGGGAAGCTCACTGG (sense primer).

GADPH: GCCTGCTTCACCACCTTCT (antisense primer).

### Western blotting

For Western blotting analysis, proteins were extracted by cell lysis in RIPA lysis buffer supplemented with PMSF protease inhibitor (1:100). The protein samples were then separated by SDS-PAGE and transferred onto nitrocellulose membranes. Following overnight incubation with primary antibodies at 4 °C, the membranes were blocked with TBST containing 5% skimmed milk for 2 h. The primary antibodies used were as follows: CGNL1 (Proteintech, 1:1000), CXCL13 (Proteintech, 1:1000), ZNF583 (BS, 1:1000), and GAPDH (CST, 1:1000). After washing with TBST, the membranes were incubated with a secondary antibody for 1 h. The protein bands were visualized using an ECL chromogenic kit (Thermo Fisher, Inc., USA) and detected using a chemiluminescence imaging system (Beijing, China).

### Statistical analysis

The data were processed, analyzed, and visualized using R software version 4.3.1. Survival analysis was conducted using the Kaplan–Meier (KM) method, and log-rank tests were performed using the survival R package. LASSO analysis with cross-validation was carried out using the glmnet R package. ROC curves were generated using the survminer R package in conjunction with the survival R package. For normally distributed variables, the Student's t-test was used, while the Wilcoxon rank-sum test was employed for variables that were not normally distributed. Statistical comparisons between two groups utilized tests such as t-tests and Mann–Whitney U tests, while one-way analyses of variance were utilized for comparisons among three groups. Data visualization was accomplished using the "ggpubr" and "ggplot2" R packages. A p-value of 0.05 was considered statistically significant for all observed differences within and between groups, unless otherwise specified.

## Results

### scRNA-Seq data preprocessing and functional analysis

Following the application of rigorous filtering criteria, which mandated that each gene be expressed in a minimum of three cells and each cell express at least 300 genes, a total of 89,471 cells were collected. Subsequently, through the assessment of mitochondrial and rRNA proportions utilizing the PercentageFeatureSet function, along with the application of additional criteria including gene expression ranging from 300 to 5000, mitochondrial content below 10%, and a minimum UMI count greater than 1000 per cell, a final dataset of 73,954 cells was obtained. Through quality analysis, we identified and excluded 0 low-quality cells. Notably, we observed a positive correlation between the number of detected genes and the total number of genes identified through sequencing.

**Figure 1:**
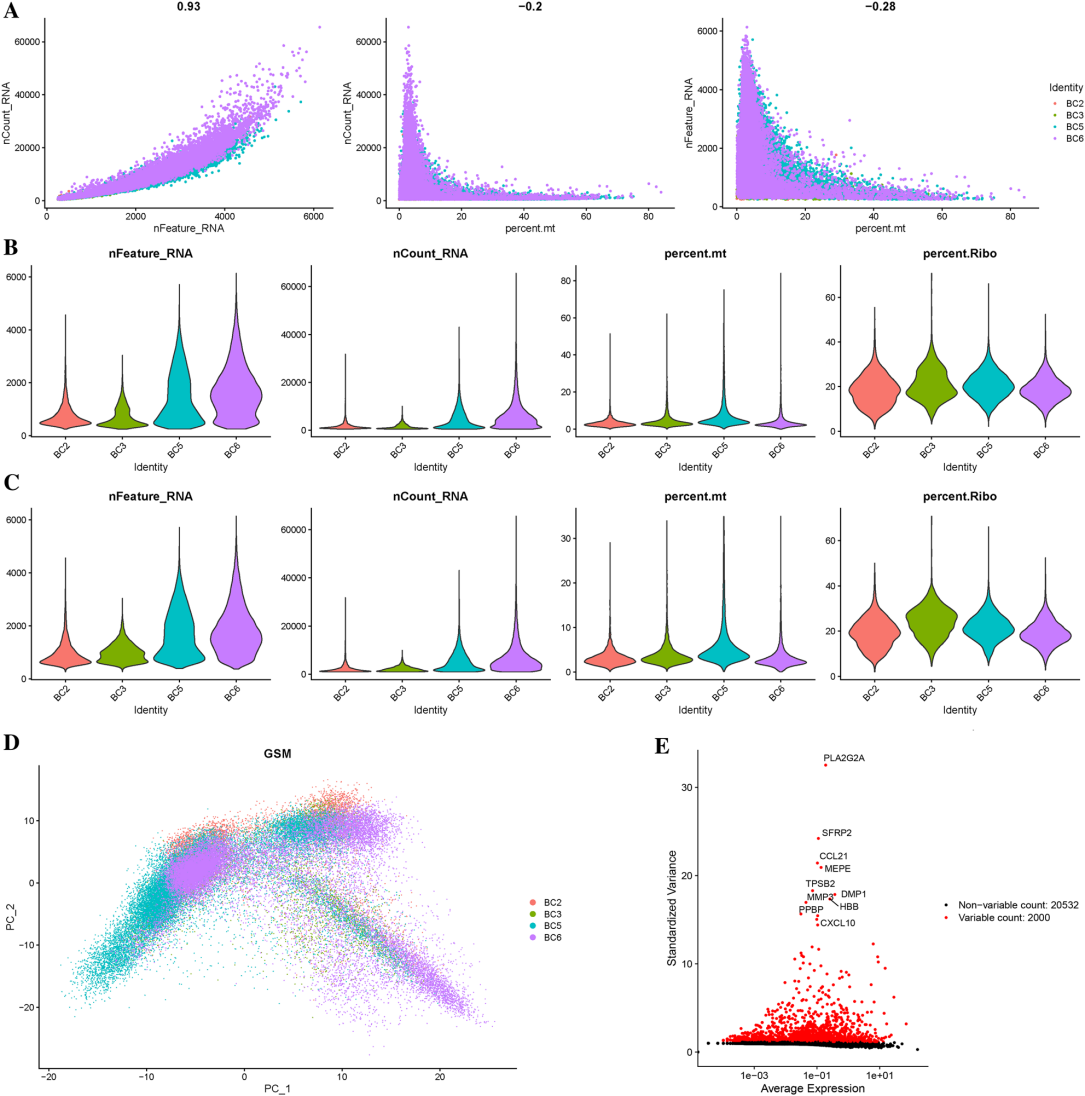
Single-cell sequencing analysis of PE and control samples. (**A**) Analysis of the correlation between nFeature and nCount, percent.mt and nCount, and percent.mt and nFeature. (**B**) Violin plots illustrating the RNA characteristic number (nFeature RNA) and absolute UMI count (nCount RNA) before quality control screening of cells. (**C**) The figure displays the total count number of each cell after quality analysis. (**D**) PCA plot. (**E**) Red dots represent the top 2000 high variation genes obtained through variance analysis.

Figure 1A illustrates a strong correlation between the number of UMI (unique molecular identifiers) and mRNA counts, while no association was found between the number of UMI/mRNA and the content of mitochondrial genes. Violin plots representing the cellular characteristics before and after quality assurance are displayed in Fig. 1B,C. By performing variance analysis on 22,532 genes, we identified the top 2000 genes that exhibited the highest variation. Among them, the top 10 genes were PLA2G2A, SFRP2, CCL21, MEPE, TPSB2, MMP3, DMP1, HBB, PPBP and CXCL10 (Fig. 1E).

**Figure 2:**
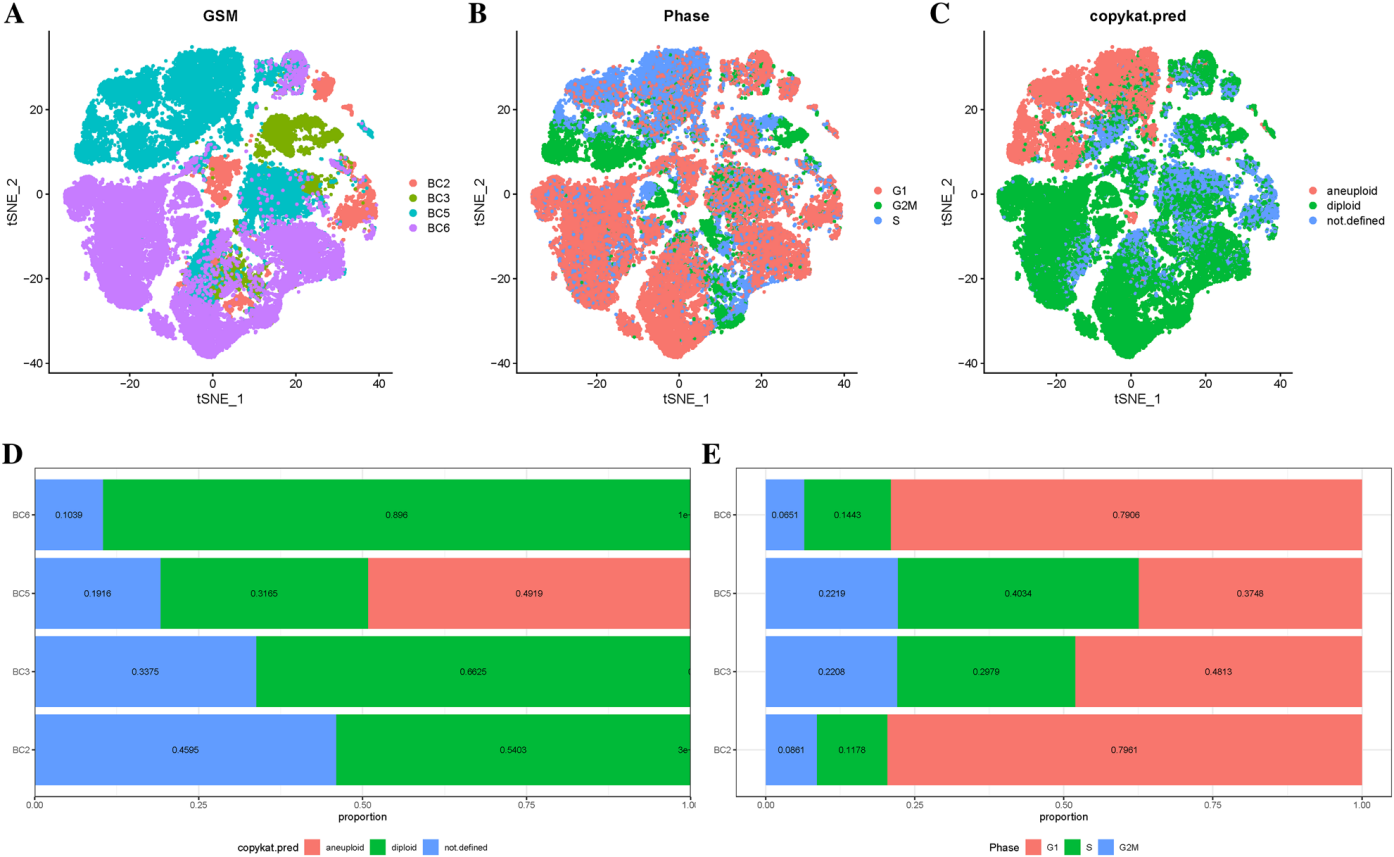
Malignant and non-malignant cells of osteosarcoma (OS) (**A**) t-SNE mapping illustrating the spatial distribution of tumor and normal cells in osteosarcoma (OS) samples, with each color representing cells from a specific sample. (**B**) t-SNE plot highlighting the distribution patterns of cell cycles, characterized by distinct colors. (**C**) t-SNE diagrams depicting the distribution of tumor and normal cells in OS samples, distinguished by different colors. (**D**) Comparative proportions of tumor and normal cells in each OS sample "diploid" refers to "normal cells", while "aneuploid" refers to "tumor cells". The term "not defined" corresponds to "undefined cells". (**E**) Proportions of cells in the G1, G2/M, and S phases in each OS sample.

To estimate the available dimensions, we employed Principal Component Analysis (PCA) and found no significant distinction between OS cells. Although the top 2000 genes underwent PCA dimension reduction, PC_1 and PC_2 did not demonstrate significant discriminatory capability among cells from different PE samples (Fig. 1D). The distribution of cells from the preeclampsia and control groups is shown in Fig. 2A. Finally, we selected 20 principal components (PCs) based on evaluation (p < 0.05) for subsequent analyses (Supplementary Fig. 1).

A TSNE dimension reduction was performed on the 45,883 cells using the RunTSNE function. The TSNE plots in Fig. 2A visualize the distribution of four samples. The CellCycleScoring function assigns scores to cells representing stages of the cell cycle based on marker genes specific to the S phase and G2M phase. Figure 2B displays the distribution of cells across different cell cycle phases. Copy number variations (CNVs) were identified using the copykat package as part of the single-cell data analysis. The results revealed 8,843 tumor cells, 28,262 normal cells, and 8,778 cells with an unknown classification (Fig. 2C). Overall, the distribution of cells across G1, G2M, and S phases, as well as the tumor cell to normal cell ratio, were examined. Figure 2D demonstrates that osteosarcoma (OS) samples have a higher proportion of normal cells than malignant cells. The G1 phase had the highest cell concentration, followed by the G2M phase. (Fig. 2E).

After identifying tumor cells in osteosarcoma (OS) tissues, we employed the single-sample Gene Set Enrichment Analysis (ssGSEA) method to calculate the enrichment scores of HALLMARK pathways at the single-cell level. Our findings revealed significant enrichment of PI3K_AKT_MTOR_SIGNALING, MTORC1_SIGNALING, and TNFA_SIGNALING_VIA_NFKB in tumor cells compared to normal cells, suggesting a potential mechanism by which tumors protect themselves and enhance survival by suppressing these processes (Supplementary Fig. 2A,B). Previous studies have investigated the involvement of NF-κB pathways, PI3K/Akt pathways and Wnt/β-catenin pathways in Anoikis regulation in tumors. Targeting MTOR and NFKB pathways may potentially induce tumor cell death and improve Anoikis in osteosarcoma, thus positively influencing patient prognoses.

### Construction of Anoikis-related molecular subclusters

After identifying 640 Anoikis-related genes, Consistent Clustering Analysis (CCA) was conducted using the "ConsensusClusterPlus" package to categorize patients based on their gene expression levels in integrated datasets. Results indicated two distinct clusters of molecular patterns: 35 patients with pattern A and 92 patients with pattern B. Principal Component Analysis (PCA) demonstrated significant differences in transcriptional patterns between these two molecular subclusters (Supplementary Fig. 3A–C). Univariate analysis was then performed to identify prognostic genes, using a significance threshold of less than 0.05 to determine gene significance. A heatmap was used to illustrate the relationship between Anoikis-related gene prognosis with gender, age, project, and molecular clusters (Supplementary Fig. 3D). Prognostic analysis for the two Anoikis-related subtypes revealed that cluster B had the best prognosis (Supplementary Fig. 3E).

### Identification of DEGs of clusters and function enrichment analysis

**Figure 3:**
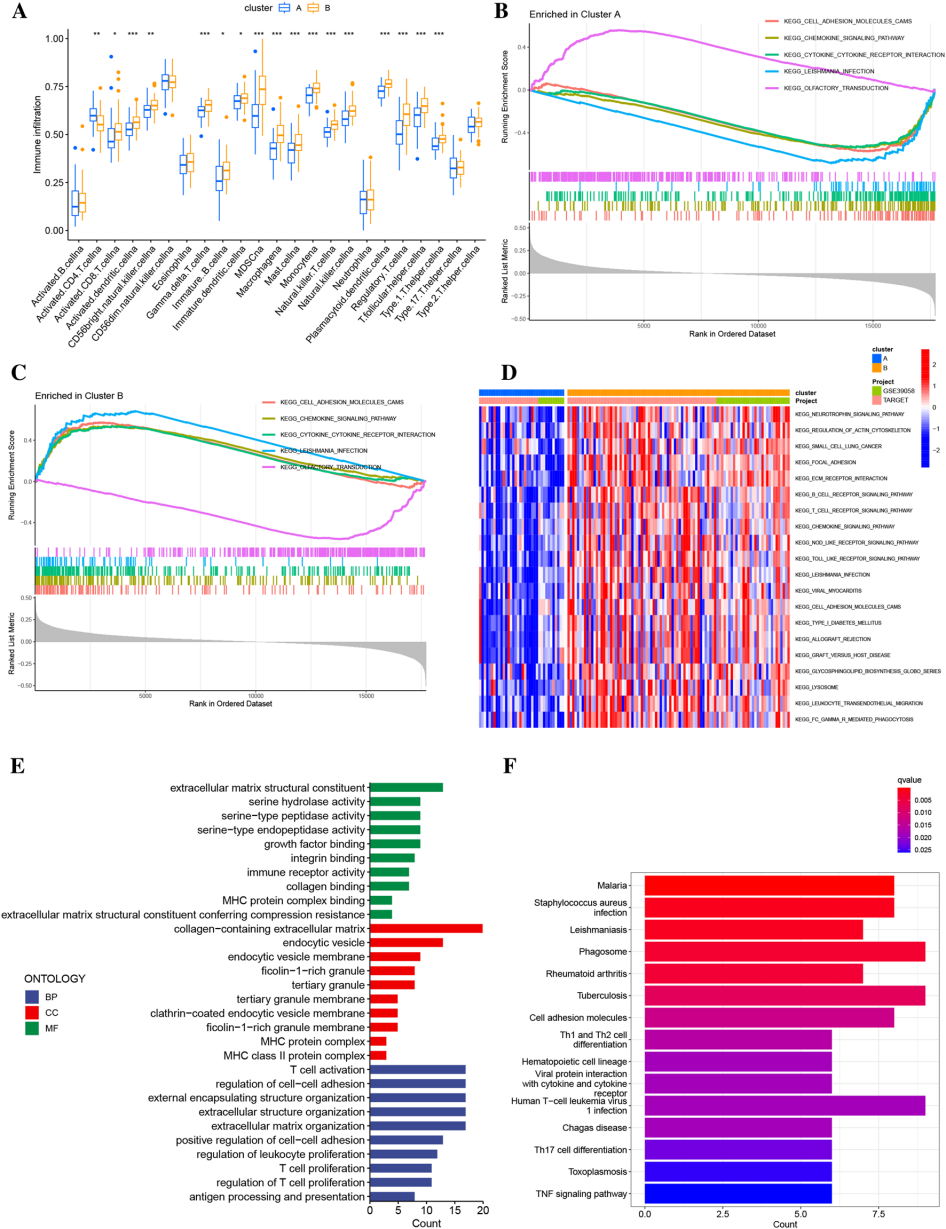
(**A**) Comparison of immune cell infiltration was performed between the two subgroups. (**B**,**C**) Gene Set Enrichment Analysis (GSEA) was conducted to identify the hallmark gene sets associated with Cluster A and Cluster B. (**D**) Gene Set Variation Analysis (GSVA) was utilized to identify the pathways associated with Cluster A and Cluster B. (**E**) The results of Gene Ontology (GO) functional enrichment analysis for Differentially Expressed Genes (DEGs) are presented. (**F**) The results of Kyoto Encyclopedia of Genes and Genomes (KEGG) pathway enrichment analysis for DEGs are also shown.

The modulation of tumor immune microenvironment (TIME) through programmed cell death can influence tumor prognosis. The infiltration of tumor-infiltrating lymphocytes (TILs) was assessed using ssGSEA analysis, which showed that Cluster B had higher levels of infiltration than Cluster A, as demonstrated in Fig. 3A. Additionally, GSEA analysis identified five biological processes, including cell adhesion molecules (CAMs), chemokine signaling pathways, and cytokine-cytokine receptor interactions, that were significantly regulated in Cluster B, as shown in Fig. 3B,C. Through the application of GSVA enrichment analysis, we investigated the biological functions of two subgroups and found that Cluster B was predominantly enriched in pathways associated with immunity, specifically those involving B cell receptor signaling and T cell receptor signaling (Fig. 3D). Additionally, signal transduction pathways related to cell adhesion, including focal adhesion, were found to be enriched in Cluster B. We identified 216 differentially expressed genes (DEGs) between Cluster A and Cluster B in order to assess differences between the two subgroups. GO analysis revealed that DEGs play a critical role in immune-related processes, such as regulation of T cell and leukocyte proliferation, T cell activation, and immune receptor activation (Fig. 3E).

Moreover, KEGG analysis revealed a higher enrichment of immune and cell adhesion-related signaling pathways in Cluster B, including Th1 and Th2 cell differentiation, cell adhesion molecules, and Th17 cell differentiation (Fig. 3F). The consensus clustering algorithm was employed to classify osteosarcoma (OS) patients into two phenotypes based on Anoikis-related gene expression modifications. However, the underlying genetic alterations and expression perturbations associated with these phenotypes remained unclear. To investigate potential transcriptional expression changes linked to Anoikis, further analysis was conducted on the two Anoikis-related modification patterns in OS. An unsupervised consensus clustering algorithm was used to analyze the 216 DEGs, leading to the identification of three genomic subtypes in the patients. Patients in gene cluster C had the poorest prognosis (23 patients) (Supplementary Fig. 4A–C). A heatmap was created to illustrate the relationships between Cluster, geneCluster, age, and project (Supplementary Fig. 4H).

### Development and validation of a model for the Anoikis subcluster-related risk score

**Figure 4:**
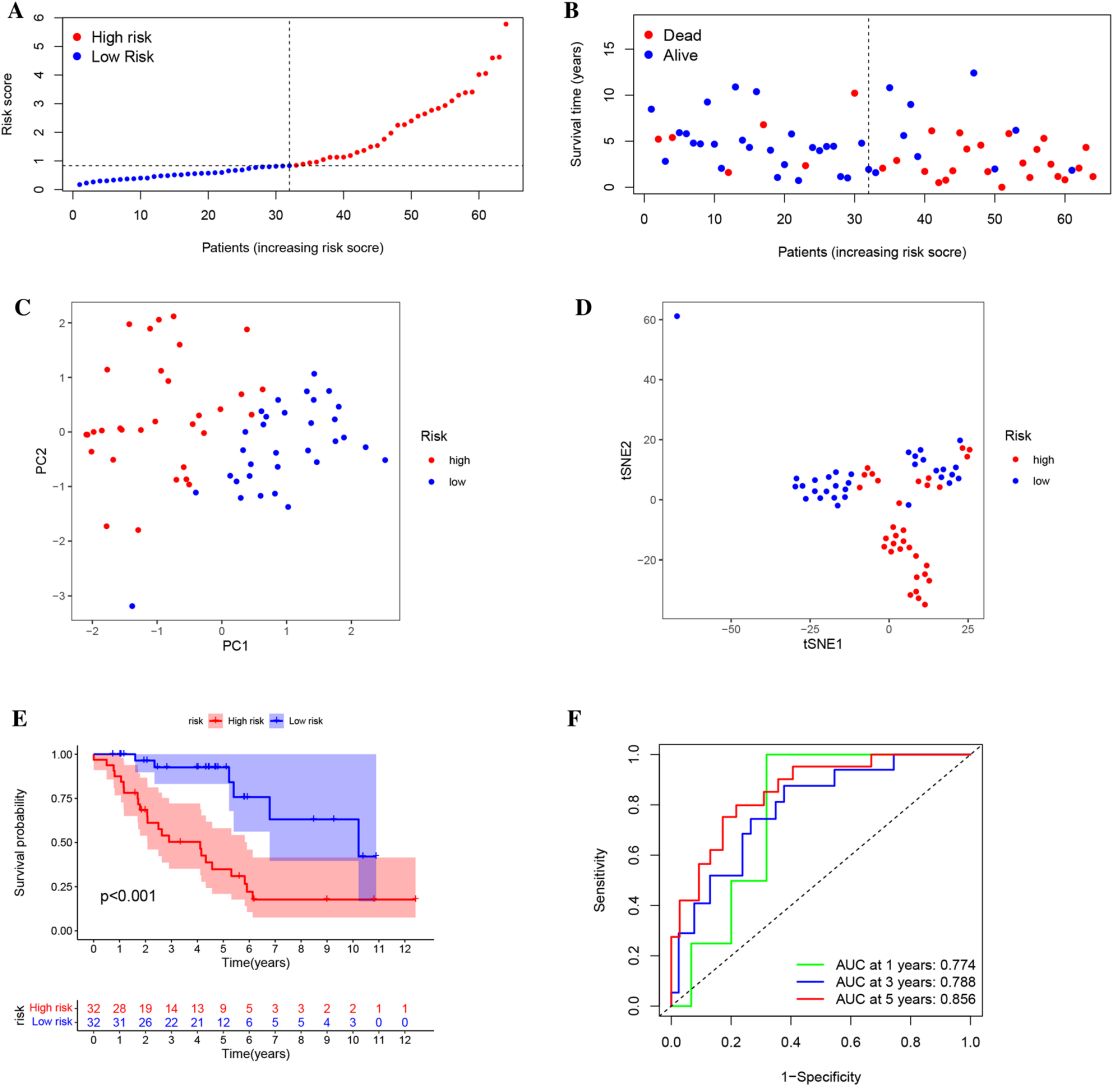
This study conducted a prognostic analysis of a 3-gene signature in the train cohort. (**A**) The risk score curve was generated to demonstrate the distribution of the model and the median score. (**B**) The distribution of survival statuses and risk scores was plotted. (**C**) Principal component analysis (PCA) was performed and a plot was generated to analyze the gene expression profiles of samples. (**D**) A t-distributed stochastic neighbor embedding (tSNE) plot was created to visualize the clustering of samples based on gene expression. (**E**) Survival analysis was performed to compare the overall survival between the two risk subgroups. (**F**) The Area Under the Curve (AUC) of the risk model was also calculated.

To identify genes linked with osteosarcoma prognosis, we performed a univariate Cox regression analysis utilizing integrated datasets from 128 patients with complete survival information. Among the 64 genes that met the p-value threshold of less than 0.05, we investigated the Anoikis subcluster-associated genes using LASSO and multivariate Cox analyses to create a robust prognosis prediction signature. We developed a prognostic signature comprised of three genes, namely ZNF583, CGNL1, and CXCL13, and employed their expression levels to calculate the riskscore. An increased riskscore was observed in both low- and high-risk groups based on median risk scores, which correlated with higher mortality (Fig. 4A,B). We employed PCA and t-SNE analyses to categorize patients into two groups using the risk score model (Fig. 4C,D), and the high-risk group showed significantly shorter overall survival (Fig. 4E, log-rank p-value < 0.001). The AUC values of ROC curves for one, three, and five years of overall survival were 0.788, 0.718, and 0.716, respectively (Fig. 4F).

Additionally, the risk score model exhibited significant variations among the three gene clusters and two Anoikis-related subtypes. The relationship between the cluster, gene cluster, and riskscore is demonstrated in Supplementary Fig. 4F–G, I. We conducted internal and external validations of the model to ensure its precision. Our results revealed an inverse correlation between the risk score for OS patients and their prognosis in survival analysis. Notably, the K-M curves for the two datasets showed a significant difference in survival rates. The ROC analysis generated AUC values of 0.792, 0.629, and 0.546 for 1-, 3-, and 5-year survival, respectively, in the internal validation group. In the external validation set, the AUC was 0.726 and 0.546 for 3- and 5-year survival, respectively (Supplementary Fig. 5). These findings suggest that the established model has excellent accuracy when applied to both internal and external validation datasets.

### Riskscore correlation with immune infiltration and prediction of immunotherapy response and sensitivity to drugs

**Figure 5:**
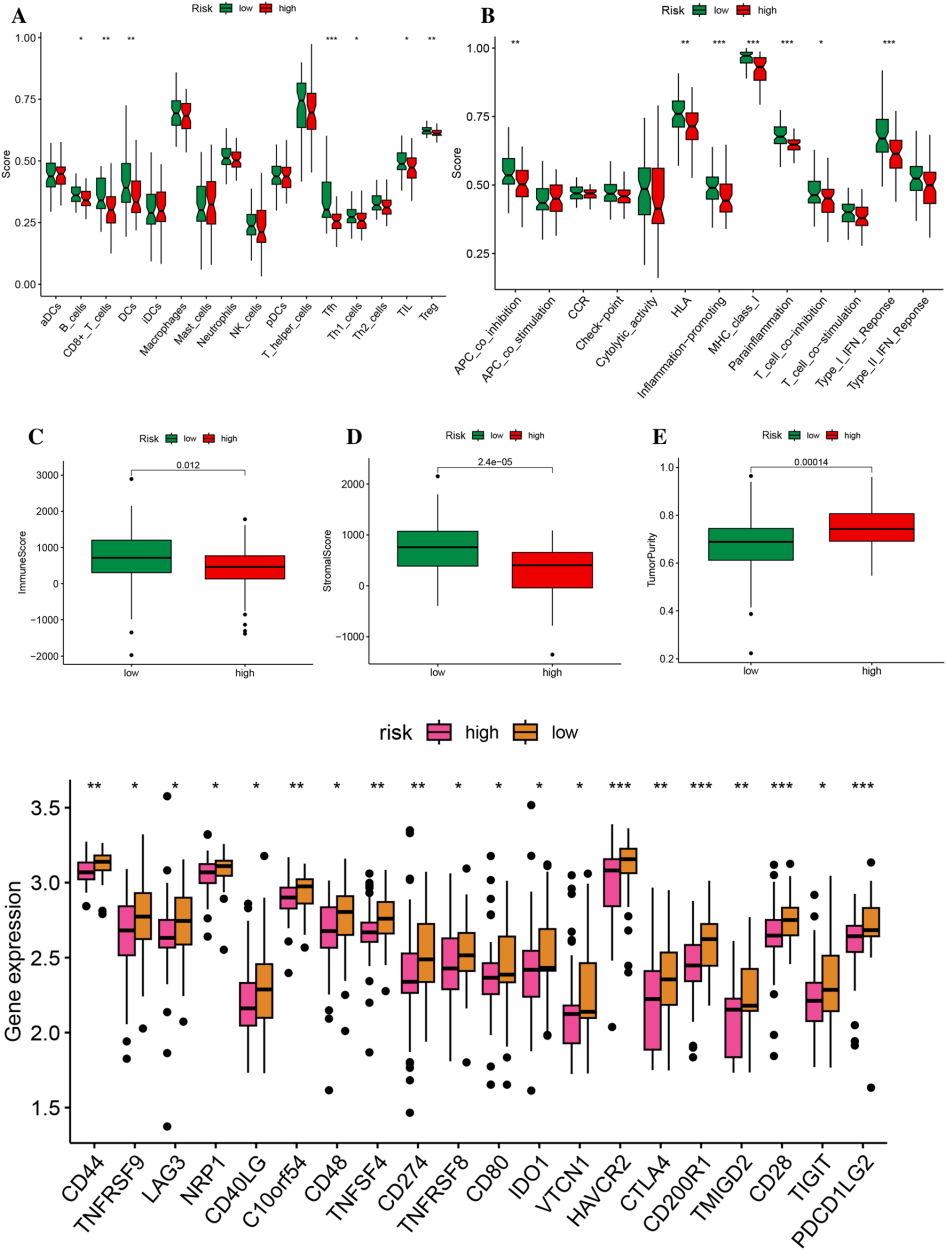
Comparison of the ssGSEA scores for immune cells and immune pathways (**A**) Differences in infiltration of 16 immune cells between high-risk and low-risk groups were calculated using the ssGSEA algorithm. (**B**) Correlations between 13 immune-related functions and the predictive signature were analyzed in high- and low-risk groups. (**C**) The Immune Score, Stromal Score, and Tumor Purity were compared between the high-risk score group and low-risk score group. (**D**) The expression of immune checkpoints was compared between the two risk subtypes. *p < 0.05; **p < 0.01; ***p < 0.001; *ns* non-significant.

To explore the association between the risk score model and immune cell fractions, such as B cells, CD8 + T cells, dendritic cells (DCs), T follicular helper (Tfh) cells, T helper 1 (Th1) cells, and regulatory T (Treg) cells, a correlation analysis was conducted. The results of the statistical analysis demonstrated a positive correlation between a lower risk score and a higher proportion of these immune cell populations (Fig. 5A,B). Additionally, the low-risk group exhibited higher immunological and stromal scores, suggesting potential immune resistance in patients (Fig. 5C,D). Furthermore, the low-risk group displayed a lower tumor cell purity, which was closely associated with higher estimated scores (Fig. 5E). Consistent with previous research, patients with an immune "hot" signature in osteosarcoma (OS) are more likely to exhibit adaptive immune resistance^17^. The expression levels of checkpoint molecules were compared between the low- and high-risk groups, which showed that the low-risk group had upregulated expression levels, suggesting the potential benefit of immune checkpoint inhibitor (ICI) treatment (Fig. 5F).

Our result reveals that ZNF583 is positively linked with activated NK cells, potentially influencing their recruitment or activation within the tumor microenvironment. CGNL1 correlates with several immune subsets, including resting CD4 memory T cells, follicular helper T cells, activated NK cells, and M2 macrophages, signifying its multifaceted role in shaping the tumor's immune landscape. Additionally, CXCL13 associates with a diverse range of immune cells, reflecting its extensive impact on tumor-associated immune responses (Supplementary Fig. 6A).

Upon analysis, it is evident that the Anoikis related riskscore distribution varies across cancer types, further emphasizing the unique characteristics of our osteosarcoma-derived gene signature. Moreover, the associations of the signature with disease-specific survival, overall survival, and progression-free interval outcomes further demonstrate its potential relevance and applicability in various oncological contexts. Notably, while the signature was derived from osteosarcoma-specific genes, its performance across other cancers underscores its broad relevance, adding value to our initial claim (Supplementary Fig. 6B).

Recent studies have suggested that combining immunotherapy with targeted therapy may increase efficacy. Several targeted drugs, including Lapatinib, Sapitinib, and Ulixertinib, have shown potential benefits for OS patients. The low-risk group was found to be more responsive to Lapatinib, Sapitinib, and Ulixertinib, while the high-risk group would benefit more from Cediranib, Crizotinib, Cyclophosphamide, Dactinomycin, Dasatinib, and Entospletinib. The study results suggest an association between riskscore and drug sensitivity (Supplementary Fig. 7C–K).

### Development and validation of the prediction model in OS

To determine a patient's prognosis, clinical characteristics play a crucial role. We developed an interactive nomogram using riskscores and clinical information such as age and gender from the TARGET dataset to predict overall survival rates for the first year, three years, and five years of the study (Supplementary Fig. 8A). The accuracy and discrimination of the prediction model were assessed using calibration curves and time-dependent ROC curves, and both curves showed the model to be reliable (Supplementary Fig. 8B,D). In addition, decision curve analysis (DCA) demonstrated that the nomogram model was more precise in predicting the 5-year survival rate (Supplementary Fig. 8C). Riskscore and health condition were identified as independent factors in the prediction model based on univariate and multivariate Cox regression analyses (Supplementary Fig. 7A,B).

### Single-cell cohort validation

**Figure 6:**
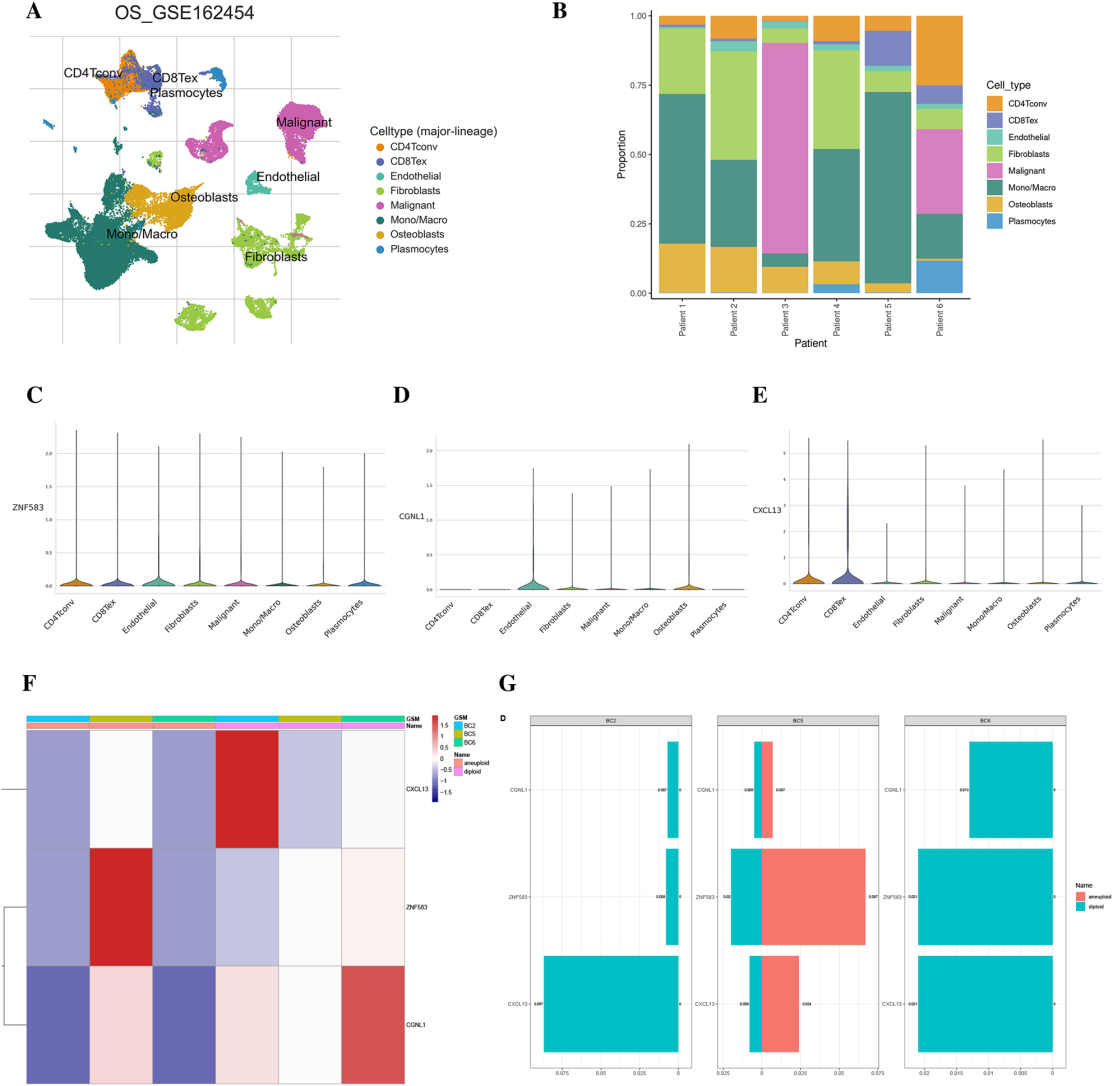
Analysis of the expression level of the three candidate genes in various cell types in GSE162454 from the TISCH database and GSE152048 from GEO dataset. (**A**,**B**) The major cell types in the dataset were identified and their proportions were annotated. (**C**–**E**) The expression of CGNL1, CXCL13, and ZNF583 was analyzed in a variety of cell types in the dataset. (**F**) Difference in expresion of three candidate genes between tumor and normal cells in OS (**G**) Expression levels of three representative genes in non-malignant and malignant cells within each osteosarcoma sample.

To examine variations in cell activity in osteosarcoma (OS), a comprehensive analysis of public single-cell sequencing data for OS was performed. Single-cell cluster analysis was carried out using TISCH2 on data from a single liver cancer cell in GSE162454 and R based on GSE152048. Subsets were annotated with different colors, and CD4Tconv cells, CD8Tex cells, endothelial cells, fibroblast cells, malignant cells, mono/macro cells, osteoblast cells, and plasmocytes were identified (Fig. 6A,B). Our findings demonstrate that ZNF583 was ubiquitously expressed in various cell types, indicating that it was not cell-specific. CGNL1 was predominantly expressed in certain osteoblasts and epithelial cells, while CXCL13 was widely expressed in T cells (Fig. 6C–E). In each sample, there is a significant difference in the expression of three genes between tumor cells and normal cells (Fig. 6F,G).

### Validation of novel genes expression levels and protein levels in cell lines

**Figure 7:**
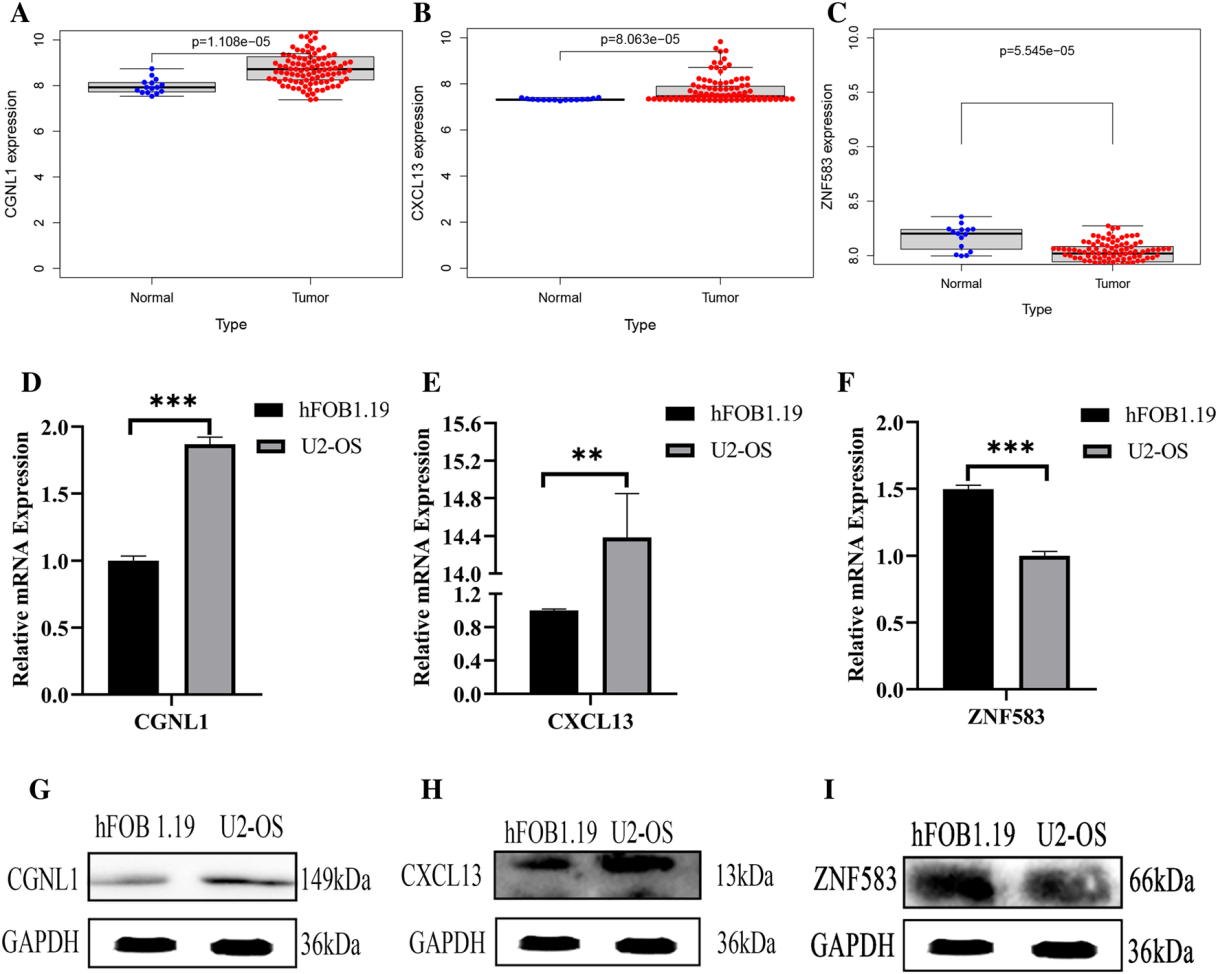
Difference expression analysis of (**A**) CGNL1, (**B**) CXCL13, (C)ZNF583 Expression of candidate genes in cell lines. (**D**–**F**) qPCR analysis (**G**–**I**) Western Blot analysis. *p < 0.05; **p < 0.01; ***p < 0.001.

To confirm the expression levels of three candidate genes, qPCR analysis was conducted on cell lines. Our qRT-PCR analysis demonstrated that osteosarcoma cells expressed higher levels of CGNL1 and CXCL13 compared to osteoblast cells, while ZNF583 had the opposite effect, indicating its potential as a novel anti-oncogene (Fig. 7D–F). Additionally, Western blot analysis was performed to evaluate the protein levels of ZNF583, CGNL1, and CXCL13. The results revealed elevated levels of CGNL1 and CXCL13 in osteosarcoma cell lines, while ZNF583 was markedly downregulated (Fig. 7G–I). These findings are consistent with the bioinformatics data (Fig. 7A–C).

### Pan-cancer analysis of ZNF583

**Figure 8:**
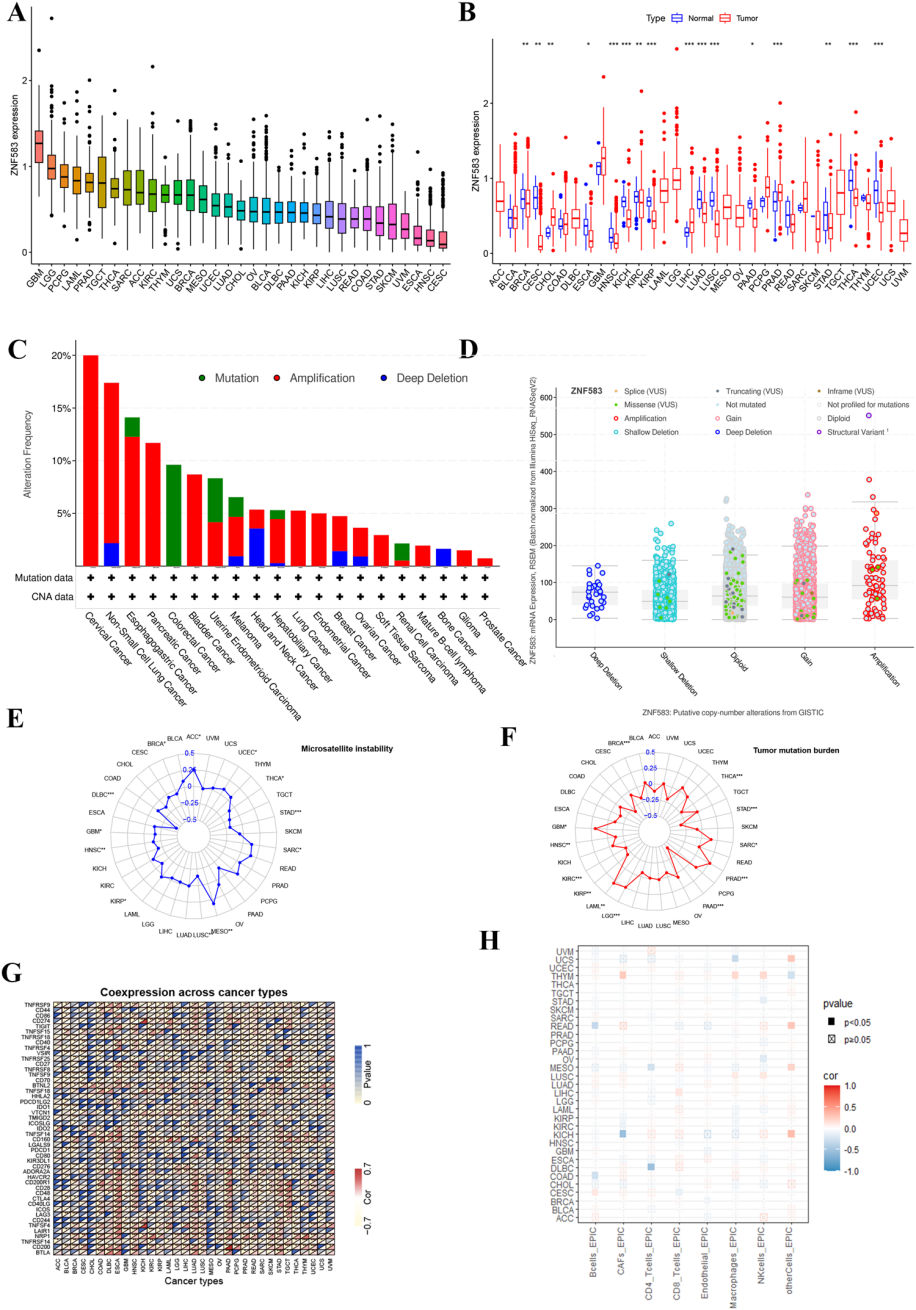
Analysis of ZNF583 in pan-cancer. (**A**) The expression levels of ZNF583 in 33 different types of cancer were analyzed. (**B**) The expression of ZNF583 was compared between tumor and normal tissue across different cancer types. (**C**) The percentage of mutations and summary of gene alterations of ZNF583 were reported across various cancers. (**D**) The relationship between ZNF583 mRNA expression and copy number alterations (CNAs) was assessed using cBioPortal. (**F**) The tumor mutation burden of ZNF583 was analyzed in pan-cancer. (**G**) The microsatellite instability of ZNF583 was analyzed in pan-cancer. (**I**) Co-expression analysis was performed to investigate the relationship between ZNF583 and immune checkpoints in pan-cancer. (**H**) Co-expression analysis was performed to investigate the relationship between ZNF583 and immune cells in pan-cancer. *P < 0.05, **P < 0.01, ***P < 0.001.

To investigate the correlation between ZNF583 and cancer progression, a thorough literature search was conducted, but only a limited number of online articles were found. To further explore its significance in other malignant tumors, a pan-cancer analysis of ZNF583 was performed. Figure 8A depicts the expression of ZNF583 in 33 types of cancer, with glioblastoma demonstrating the highest levels of expression. When compared with normal paracancerous tissues, several cancer types exhibited significant variations in ZNF583 expression (Fig. 8B). The majority of human malignancies stem from mutations in the human genome. Accordingly, we utilized the cBioportal database to investigate the mutant landscape of ZNF583 in diverse types of cancer. Our findings indicate that ZNF583 amplification was the primary type of alteration present in cervical cancer patients, while mutational copy number alteration (CNA) and copy number deep deletion were predominant in colorectal cancer and head and neck cancer, respectively (as depicted in Fig. 8C–E). Upon conducting further analysis, it became apparent that the mutation rate and types of ZNF583 varied across different tissues and diseases. Furthermore, ZNF583 has been associated with tumor mutation burden (TMB) and microsatellite instability (MSI) in different cancer types, as shown in Fig. 8F,G. To investigate the relationship between ZNF583 and immune-related genes, as well as immune checkpoints, a gene co-expression analysis was performed. The findings depicted in Fig. 8H–I suggest that ZNF583 may influence immune cell infiltration and the expression of immune checkpoints across multiple cancer types.

## Discussion

Osteosarcoma is a highly invasive malignant tumor that significantly contributes to the unfavorable prognosis observed in most affected patients^18^. Consequently, it is crucial to develop and explore prognostic models for osteosarcoma to identify targeted therapies. Various researchers have utilized bioinformatics and sequencing technologies to investigate the characteristics of osteosarcoma and develop prognostic models. It is worth noting that the process of Anoikis, which induces apoptosis through detachment from the extracellular matrix, plays a crucial role in the tumor's growth and metastasis^19^. Several studies have consistently demonstrated that the activation of downstream signaling pathways, such as PI3K/Akt, plays a significant role in promoting resistance to anoikis, particularly in osteosarcomas. The resistance of osteosarcoma cells to anoikis can be increased by promoting angiogenesis and tumor invasion through the Src/JNK/ERK signaling pathway, which leads to increased VEGF-A expression^20^.

The aim of this research was to assess the relevance of Anoikis-associated genes in osteosarcoma by conducting an extensive analysis of both single-cell RNA sequencing and bulk RNA sequencing data. Following quality control and normalization procedures for the scRNA-seq data, the individual cells were categorized into two distinct subpopulations: aneuploid cells and diploid cells.

In addition to well-known cell death mechanisms such as apoptosis and pyroptosis, emerging evidence suggests the involvement of a novel form of Anoikis that holds promise for improving patient prognosis. Consistent with these findings, our study indicates that aneuploid cells may evade cell death by suppressing Anoikis activities when compared to diploid cells. These results provide compelling evidence for the protective role of Anoikis in OS, with tumor cells potentially employing the downregulation of Anoikis activities as a self-protective mechanism.

To elucidate the underlying prognostic mechanism of the proposed Anoikis-based molecular subtype, a consensus clustering approach was utilized to stratify osteosarcoma patients into two distinct subtypes based on Anoikis-related genes. Prognostic analysis revealed that cluster A exhibited the poorest prognosis among the identified subtypes. The majority of lymphocytes infiltrating the tumors consisted of activated CD4 and CD8 T cells, which exhibited significant differences in immune infiltration. Our findings indicate that the proportion of Tregs is elevated in Cluster B. Notably, Tregs are traditionally viewed as an immune tolerance subset, known to suppress cytotoxic T cell activities within the tumor microenvironment. Within Cluster B's milieu, the elevated Treg ratio might extend beyond traditional immune dampening, possibly aiding a balanced immune response and influencing the tumor cells, leading to improved prognosis. Our analysis suggests that Tregs in Cluster B might possess a unique functional signature. They could modulate excessive inflammation, curbing tissue damage and fostering a more potent antitumor immune reaction. Moreover, the interplay between Tregs and other immune factions in Cluster B's tumor environment could exhibit a distinct regulatory synergy conducive to tumor suppression over its growth^21^. Tregs, by secreting IL-10 and TGF-β1, inhibit osteoclast differentiation and bone resorption, potentially exerting specific effects on tumor cells, culminating in a more positive prognosis^22^. To delve deeper into the functional differences between the two clusters, gene set variation analysis (GSVA) was performed, revealing that cluster B exhibited a notable enrichment of immune-related pathways and adhesion pathways, including focal adhesion. Focal adhesions, acting as connectors between tumor cells and the extracellular matrix, have been demonstrated in previous studies to play a critical role in tumor migration, invasion, and drug resistance^23^, which confirmed prognosis differences in the 2 subtypes. Following differential analysis between the two subtypes, a total of 216 genes were identified as differentially expressed genes. GO and KEGG enrichment analyses conducted on these DEGs revealed a significant enrichment of cell adhesion-related signaling pathways. This finding suggests a close association between the key Anoikis genes within the module and the process of metastasis.

Univariate Cox and LASSO Cox regression analyses of the significant Anoikis subtype-related genes (ZNF583, CGNL1, and CXCL13) identified three genes relevant to constructing the Anoikis Subcluster-related genes signature. The signature demonstrated good predictive ability in all cohorts, with AUC values of 0.788, 0.718, and 0.716 at 1, 3, and 5 years, respectively. Furthermore, the riskscore can serve as an independent prognostic indicator for osteosarcoma patients.

The tumor microenvironment (TME) comprises immune cells, stromal cells, and tumor cells, all of which impact tumor progression and prognosis. The antitumor effect is more pronounced when immune cells are present between tumor cells rather than stromal cells. The innate and adaptive immune systems collaborate to identify and eradicate tumor cells, with distinct roles played by various immune cell types, including CD4 + T-helper cells (e.g., TH1, TH2, and TH17 cells), CD8 + cytotoxic T cells, γδT cells, natural killer (NK) cells, and natural killer T (NKT) cells^24^. The low-risk group exhibited significantly higher expression levels of B cells, CD8 + T cells, dendritic cells (DCs), and Th1 cells when compared to the high-risk group. Additionally, it has been suggested that natural killer T (NKT) cells play a crucial role in inhibiting tumor cell proliferation and influencing the clinical outcomes of cancer patients^25^. A positive correlation has been established between increased infiltration of tumor-associated macrophages and a more favorable prognosis in certain malignant tumors^26,27^. The correlation between decreased riskscores, increased immunity, and a higher immune score was observed, indicating that riskscore may be a means of evaluating the immunocompetence of osteosarcoma (OS) patients and predicting immunotherapy response. Patients with lower riskscores had higher expression rates of immune checkpoint molecules and improved overall survival rates, which suggests that they are more suitable for immune checkpoint inhibitor (ICI) therapy. Risk scores can assist in classifying patients into low- and high-risk groups, and ICIs have been shown to be effective and safe. Furthermore, our study discovered that 10 immune checkpoint genes exhibited differential expression between the two groups, with the low-risk group demonstrating higher expression rates. This finding may indicate that the efficacy of immunotherapy is limited. The results of our study are consistent with the observation that patients with low-risk scores have higher survival rates.

According to our drug sensitivity analysis, Lapatinib, Sapitinib, and Ulixertinib were found to be more effective for patients with lower riskscores, while Cediranib, Crizotinib, Cyclophosphamide, Dactinomycin, Dasatinib, and Entospletinib were more suitable for those with higher riskscores. This information can assist in clinical decision-making regarding the selection of immunotherapy and targeted therapy combinations. Our study highlights the utility of riskscore in identifying osteosarcoma (OS) patients who are likely to benefit from immunotherapy and predicting their prognosis based on riskscore expression.

In light of personalized osteosarcoma treatment, the riskScore we've introduced becomes paramount. By categorizing patients based on this score, we can precisely stratify patients into different risk groups, each potentially requiring a distinct therapeutic approach. Higher riskScore individuals, for instance, may necessitate aggressive treatment modalities or novel agents that specifically target the identified genes in our study. On the other hand, those with lower riskScores might benefit more from immunotherapies, given the heightened expression of immune checkpoint molecules. This kind of precision in patient stratification ensures that treatment modalities are not 'one-size-fits-all', but are tailored to each patient's unique genomic landscape. As the medical community continues its stride toward individualized care, such riskScore analyses will be pivotal in delivering the most efficacious therapeutic strategies for osteosarcoma patients.

CGNL1 is present in both adherens and tight junctions. It functions in epithelial cells by activating Rac1 through GEF Tiam1 and suppressing RhoA activity. CGNL1 regulates Rho family GTPases by bringing guanine nucleotide exchange factors to epithelial junctions, and it also plays a role in GTPase-mediated angiogenesis^28^. Initially identified as a chemotactic factor for B-cells, CXCL13 has been demonstrated to have an essential function in immune system function and the development of inflammatory bowel disease^29^. The interaction of CXCL13 with its corresponding G-protein coupled receptor (GPCR), CXCR5, is essential not only for normal physiological processes but also for the development of inflammatory disorders and neoplastic conditions^30^. Research has demonstrated that the CXCL13/CXCR5 axis activates signaling pathways such as phospholipase C beta (PLC), protein kinase C (PKC), c-Src, and nuclear factor-κB (NF-κB) to induce cell migration and stimulate the expression of vascular cell adhesion molecule-1 (VCAM-1) in human osteosarcoma^31^. A decrease in Zinc finger protein 583 (ZNF583) expression has been observed in relation to chemoradiotherapy resistance in esophageal cancer^32^, and ZNF583 hypermethylation may also mark tumors with a poor prognosis or therapeutic response^33^. Furthermore, few publications have examined the correlation between ZNF583 and cancer. In our study, we observed notable variations in ZNF583 protein expression during experimental validation between osteosarcoma and human osteoblast cell lines.

A thorough investigation was carried out to explore ZNF583 expression in various cancer types, aiming to comprehend its involvement in cancer. ZNF583 was predominantly found to be downregulated in most of the examined cancers. Furthermore, ZNF583's role in tumor microenvironment (TME), microsatellite instability (MSI), immune cell infiltration, and immune checkpoint regulation was also observed in pan-cancer settings, in addition to its involvement in osteosarcoma progression.

Despite the strengths of this study, there are notable limitations. The number of samples retrieved from the GEO or TARGET databases was limited, which may impact the comprehensiveness of the proposed model and the clarification of potential mechanisms. The lack of clinical information, such as tumor grade, may have relevance to osteosarcoma prognosis. To evaluate the clinical utility of the findings, a larger, multicenter, prospective clinical cohort study will be necessary. Implementing our osteosarcoma gene signature clinically faces challenges like robust validation due to genetic diversity and integration into existing treatment protocols. Ethical issues surrounding genetic data and patient consent also warrant attention. Despite these, the signature’s potential in personalized treatment is significant. Additionally, further validation studies are required to confirm the accuracy of the study, including noncoding RNAs such as lncRNAs and microRNAs that play crucial roles in osteosarcoma tumorigenesis, progression, and immunity.

## Conclusion

A novel prognostic signature pertinent to osteosarcoma has been elucidated. Our findings underscore the pivotal role of Anoikis in osteosarcoma pathogenesis. This signature bears profound clinical ramifications, paving the way for personalized therapeutic regimens. Its capacity to steer targeted therapeutic interventions and shape subsequent research endeavors to enhance osteosarcoma patient outcomes is noteworthy.

### Supplementary Information

Supplementary Information 1.

Supplementary Figure 1.

Supplementary Figure 2.

Supplementary Figure 3.

Supplementary Figure 4.

Supplementary Figure 5.

Supplementary Figure 6.

Supplementary Figure 7.

Supplementary Figure 8.

Supplementary Information 10.

Supplementary Legends.

## Data Availability

Data generated or used in this study are available from the corresponding authors upon reasonable request.
