# Supplementary figures and images for: Single-cell and bulk RNA sequencing reveals Anoikis related genes to guide prognosis and immunotherapy in osteosarcoma
Source: Sci Rep. 2023 Nov 18;13:20203. doi: 10.1038/s41598-023-47367-3 (PMC10657454; doi:10.1038/s41598-023-47367-3)

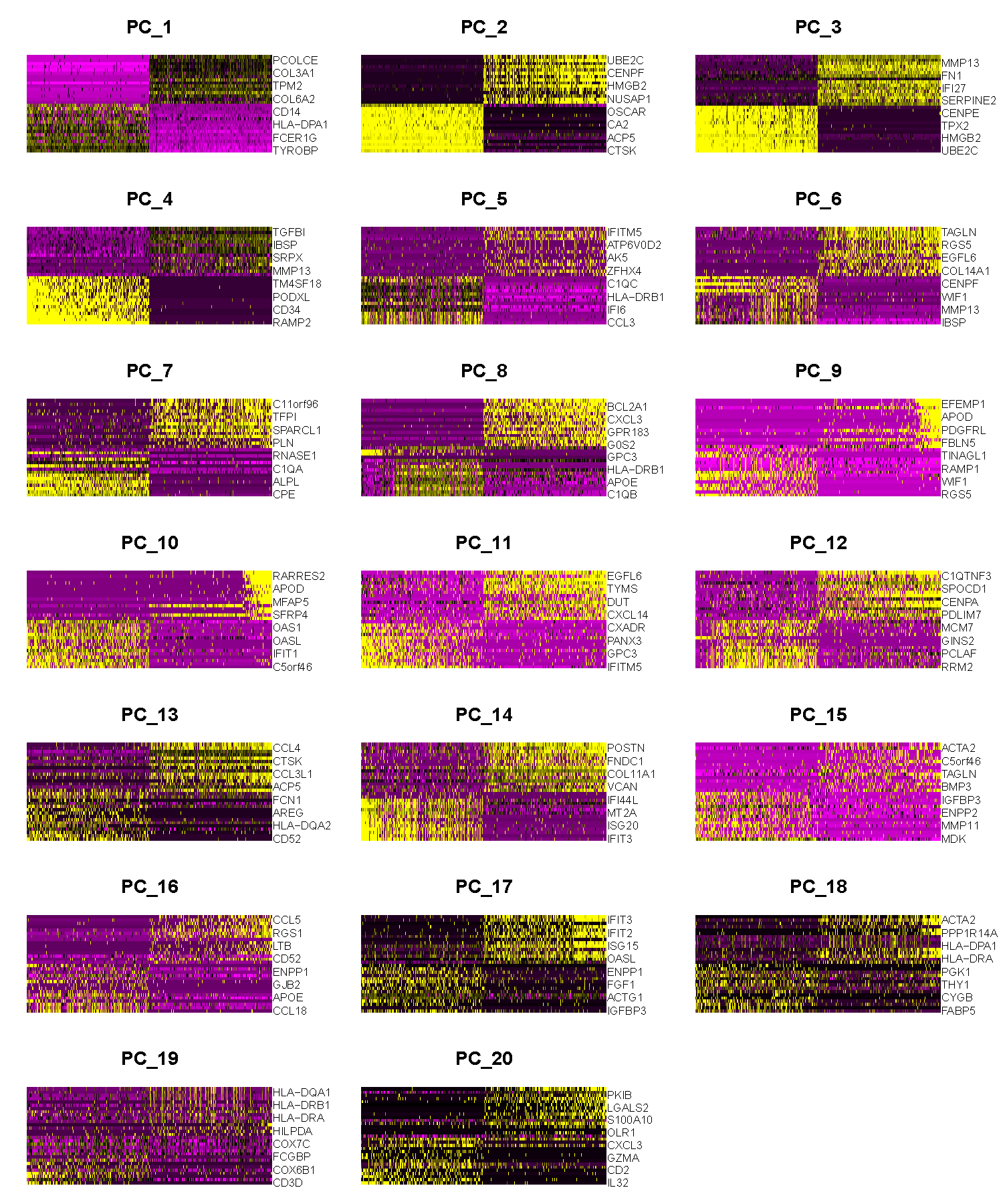

Supplement: Supplementary file 2 — Supplementary Figure 1. [file 41598_2023_47367_MOESM2_ESM.tiff]

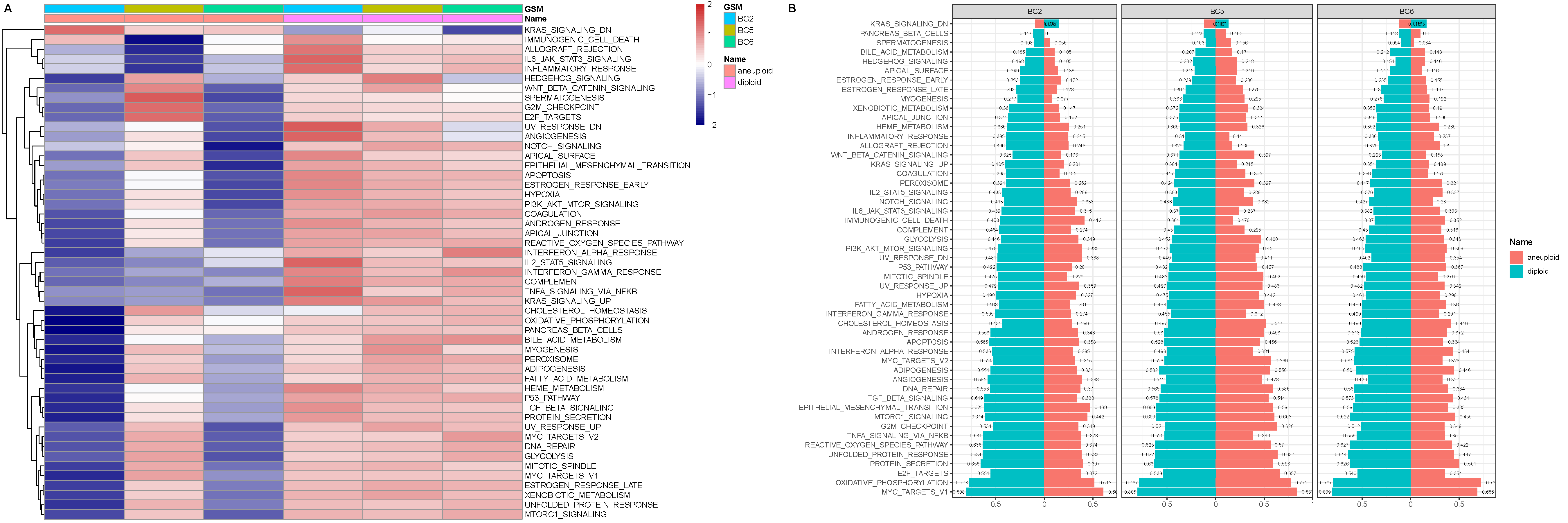

Supplement: Supplementary file 3 — Supplementary Figure 2. [file 41598_2023_47367_MOESM3_ESM.tiff]

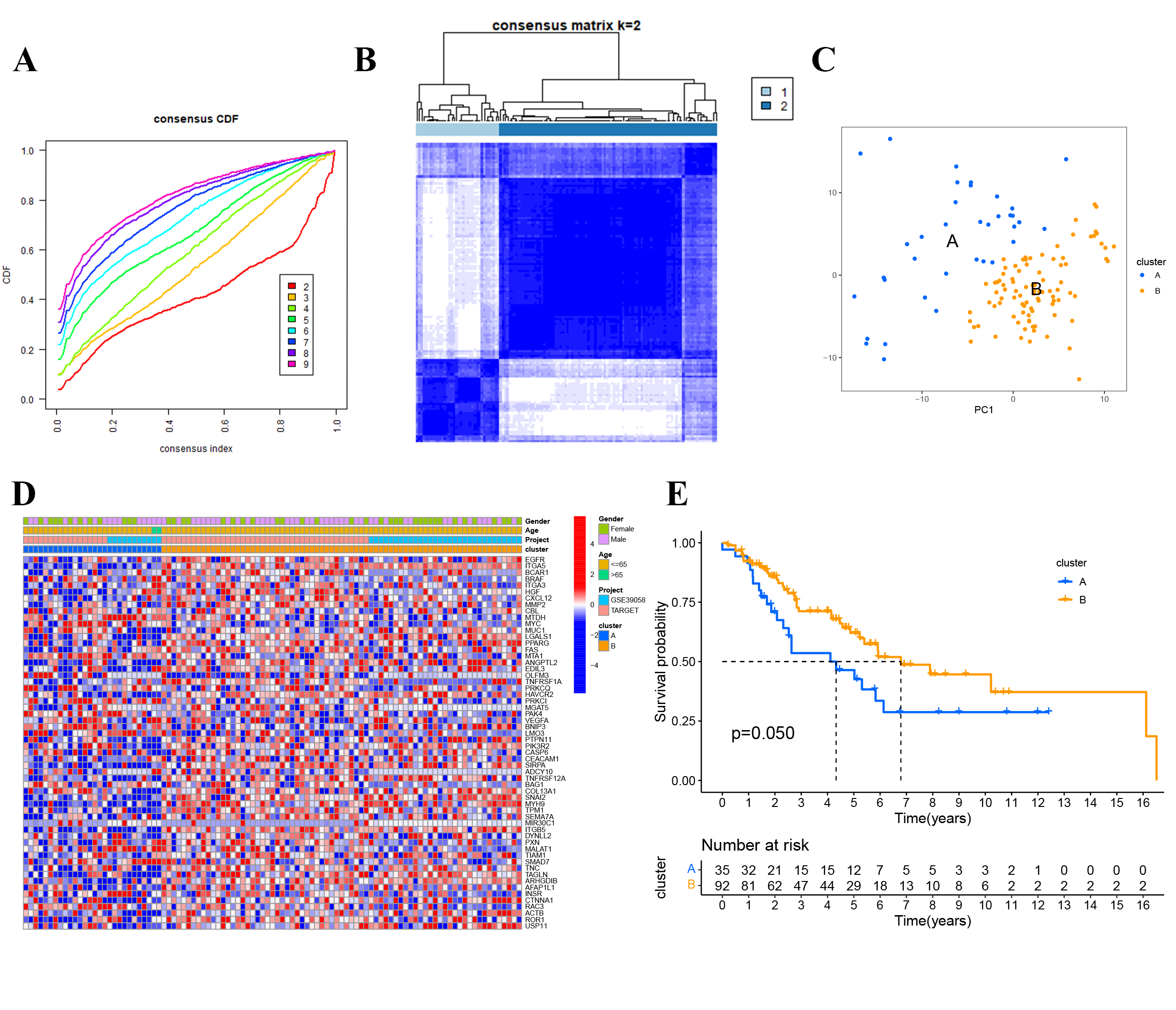

Supplement: Supplementary file 4 — Supplementary Figure 3. [file 41598_2023_47367_MOESM4_ESM.tif]

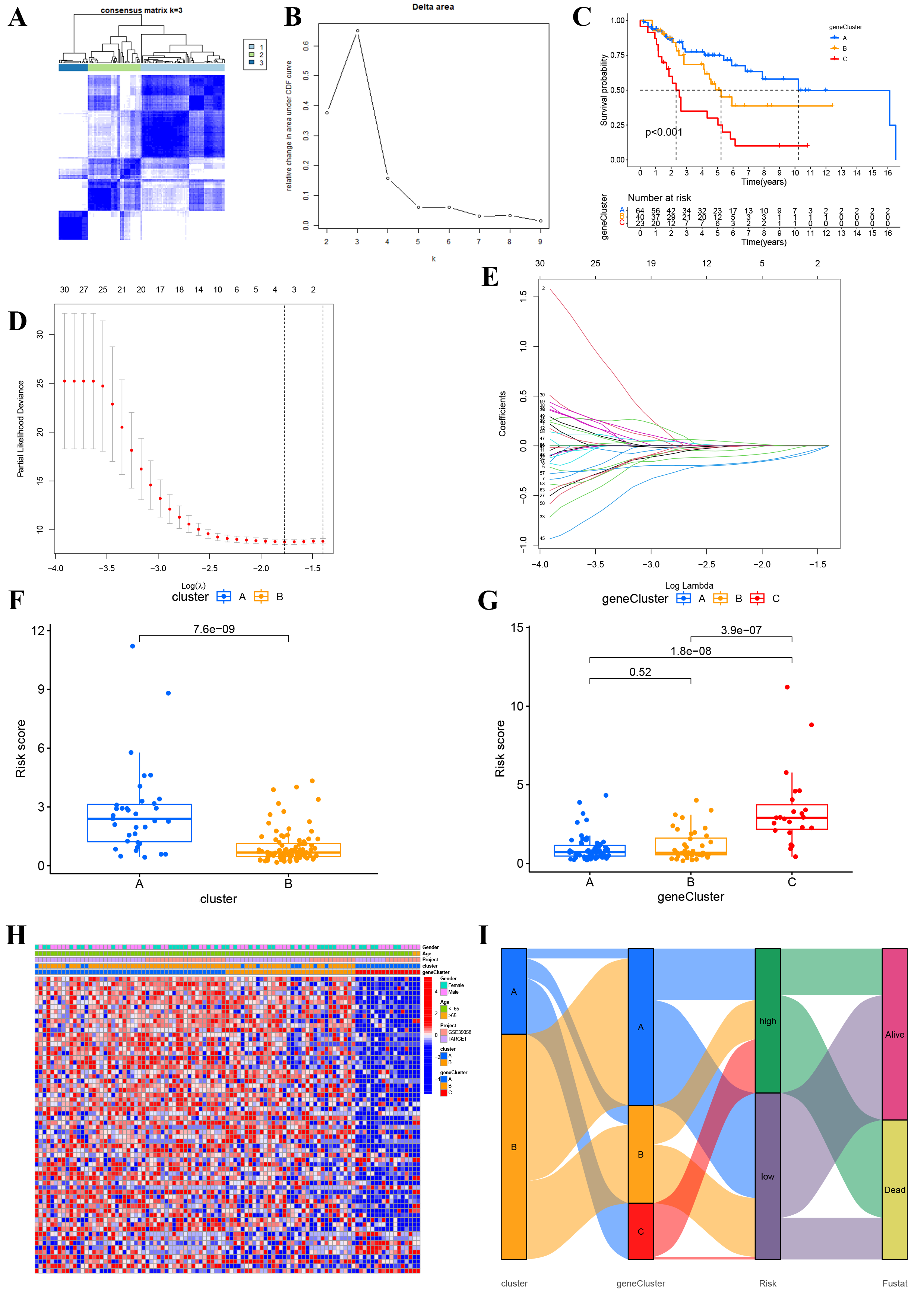

Supplement: Supplementary file 5 — Supplementary Figure 4. [file 41598_2023_47367_MOESM5_ESM.tif]

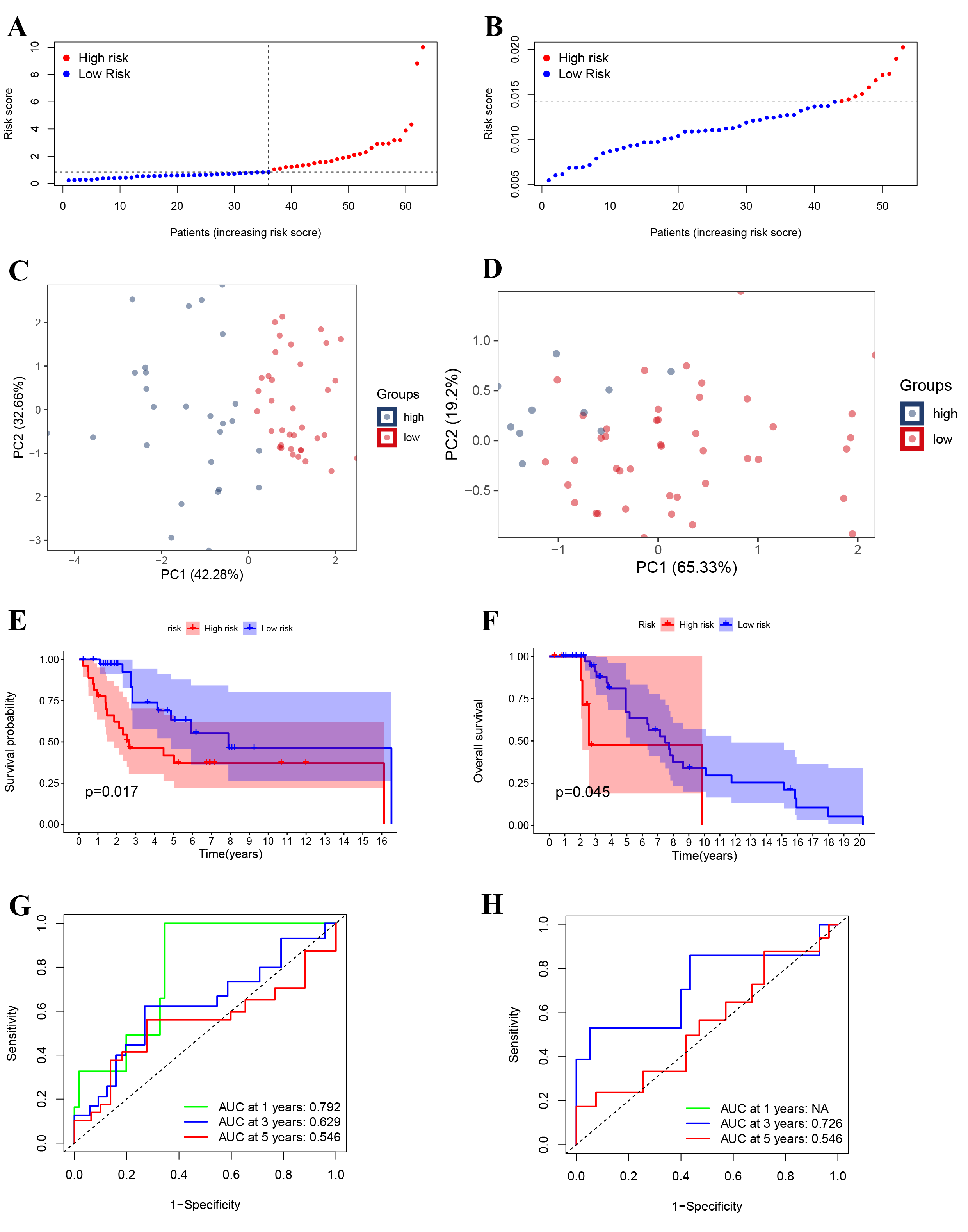

Supplement: Supplementary file 6 — Supplementary Figure 5. [file 41598_2023_47367_MOESM6_ESM.tif]

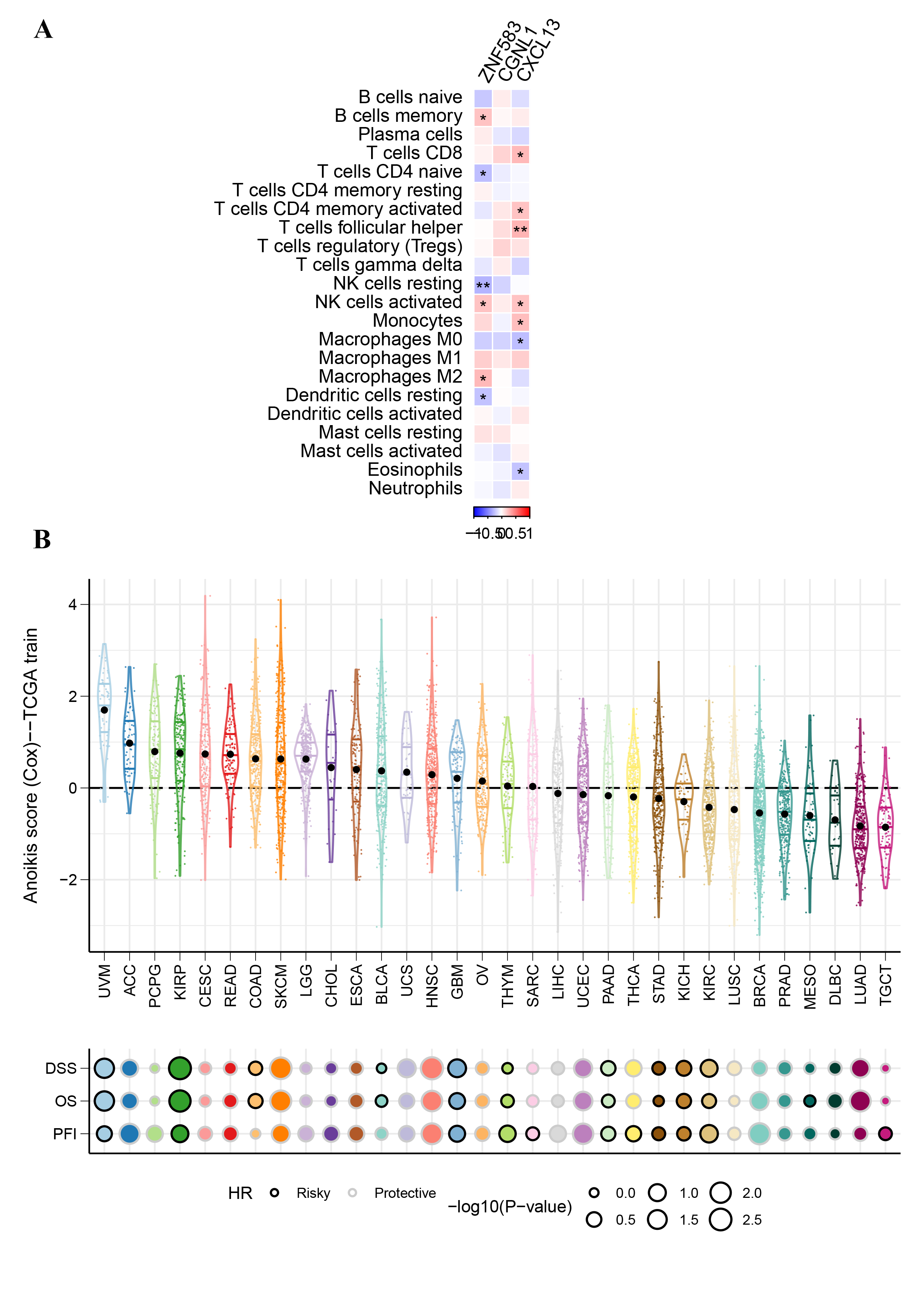

Supplement: Supplementary file 7 — Supplementary Figure 6. [file 41598_2023_47367_MOESM7_ESM.tif]

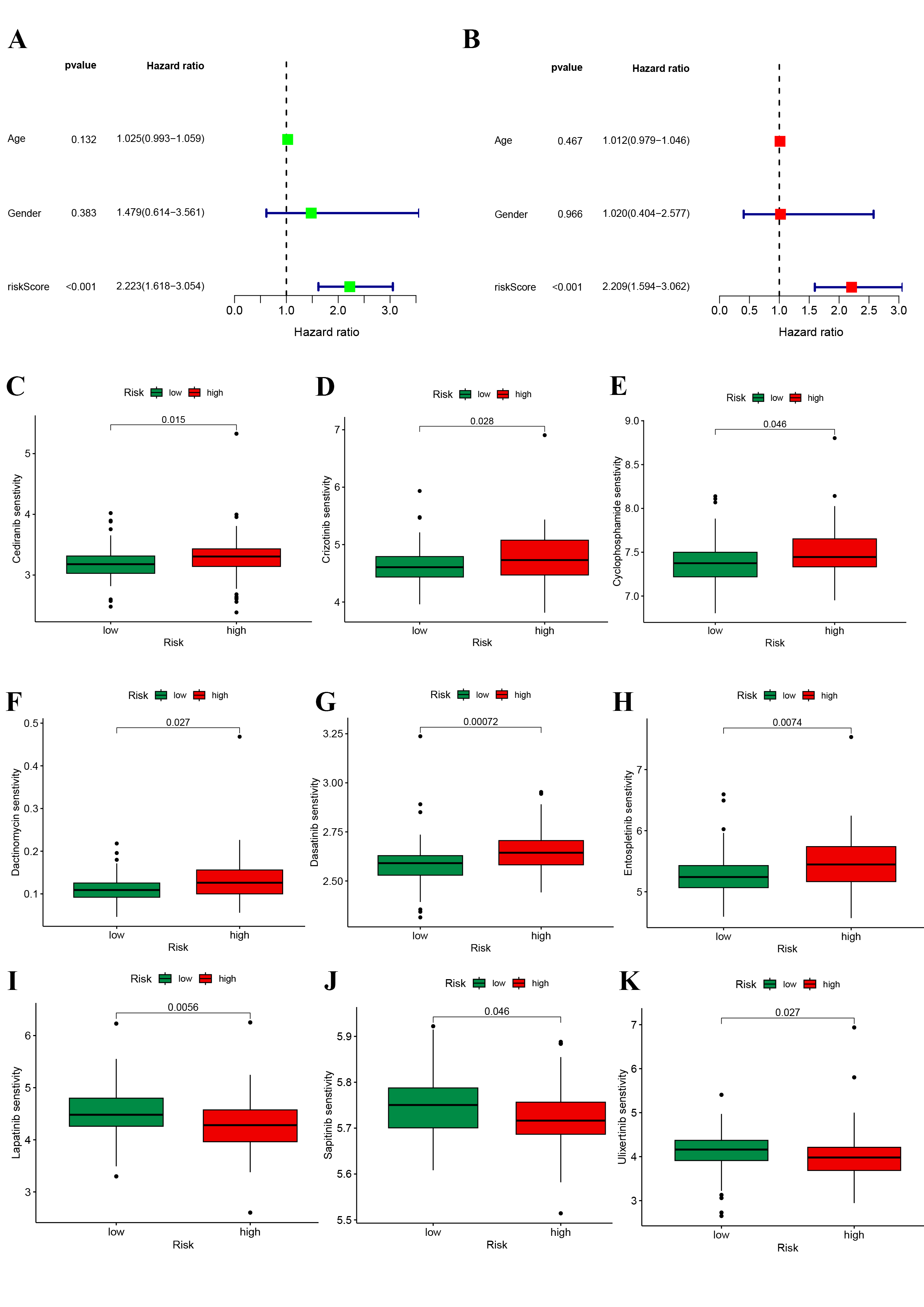

Supplement: Supplementary file 8 — Supplementary Figure 7. [file 41598_2023_47367_MOESM8_ESM.tif]

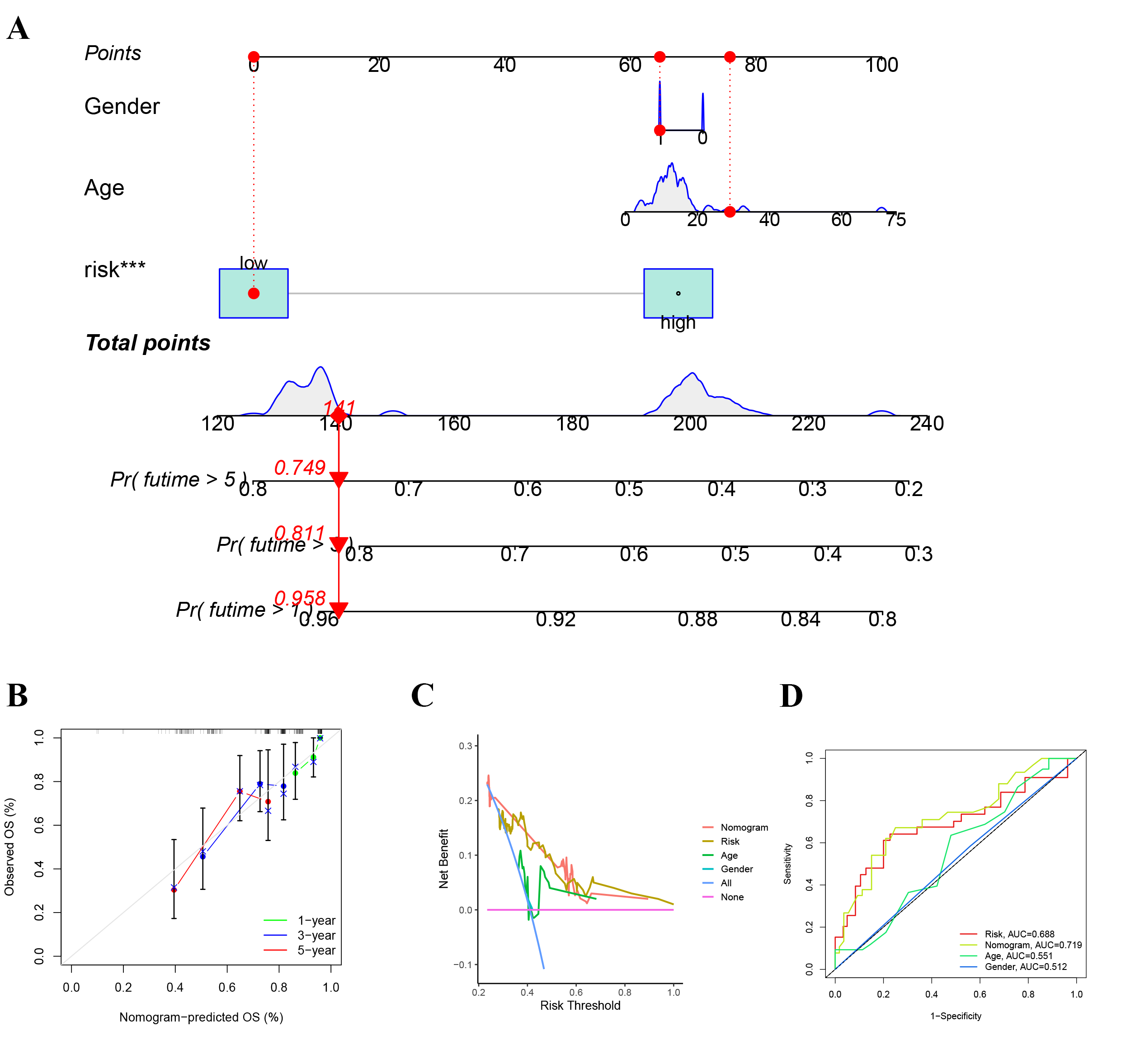

Supplement: Supplementary file 9 — Supplementary Figure 8. [file 41598_2023_47367_MOESM9_ESM.tif]

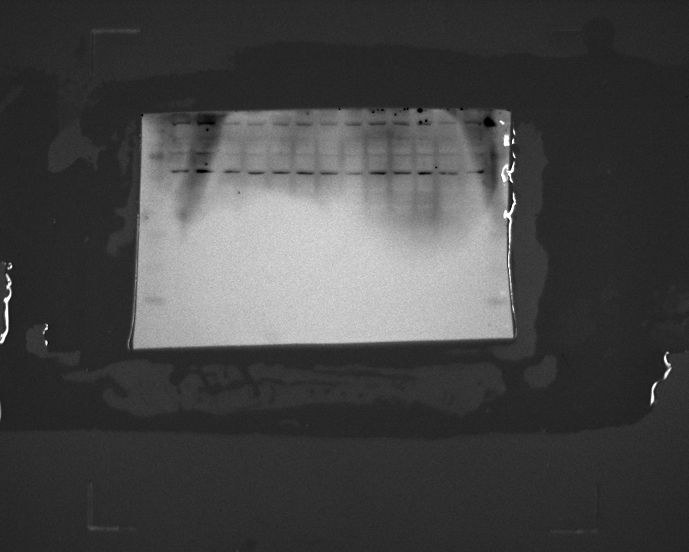


CGNL1


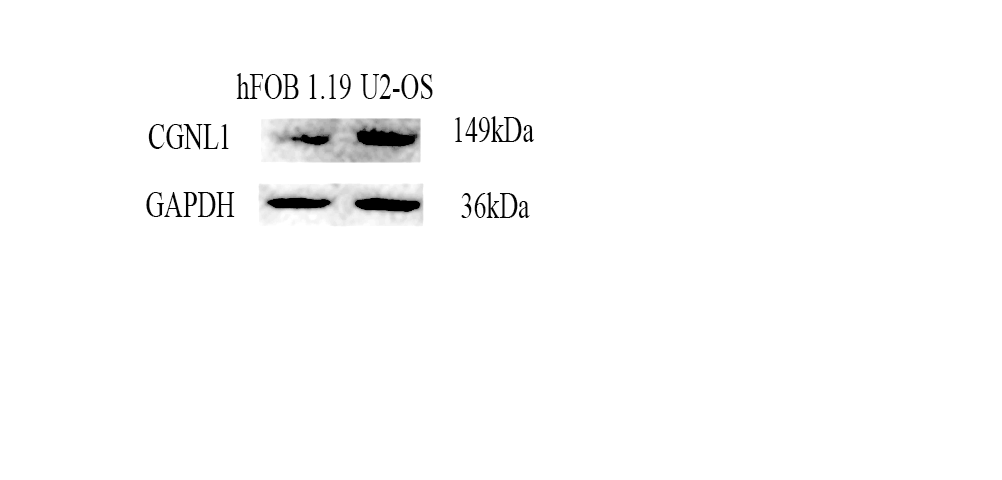


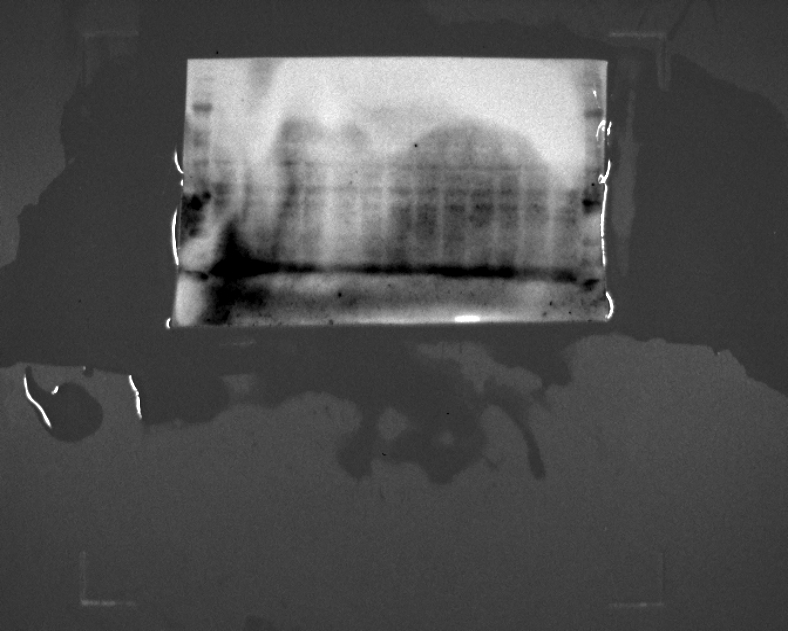


CXCL13


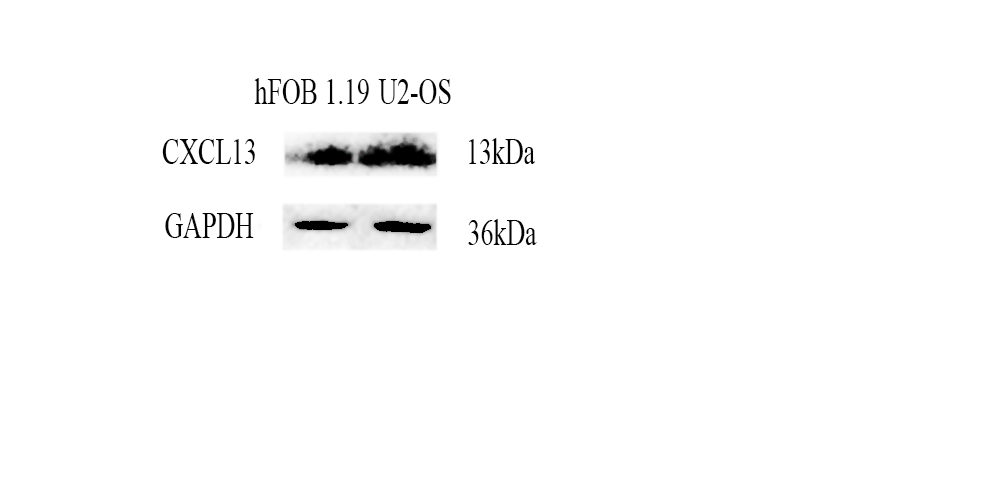

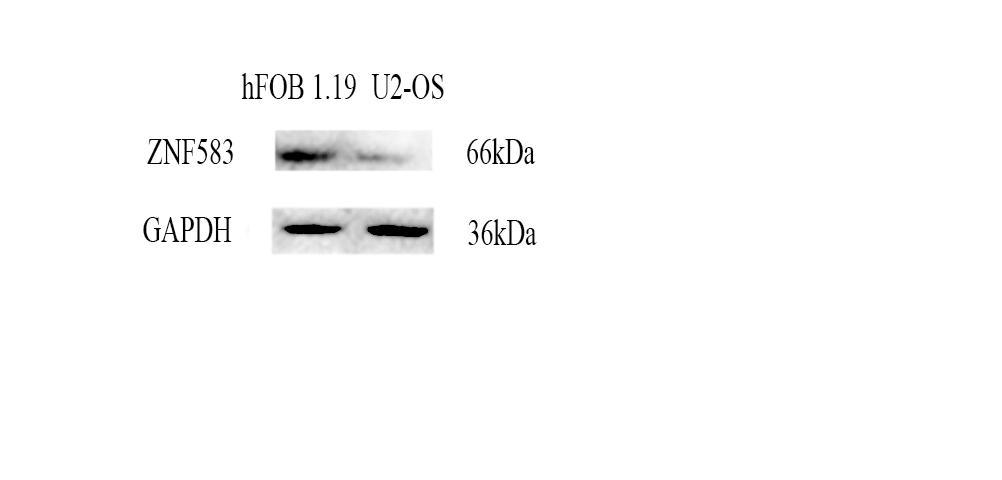


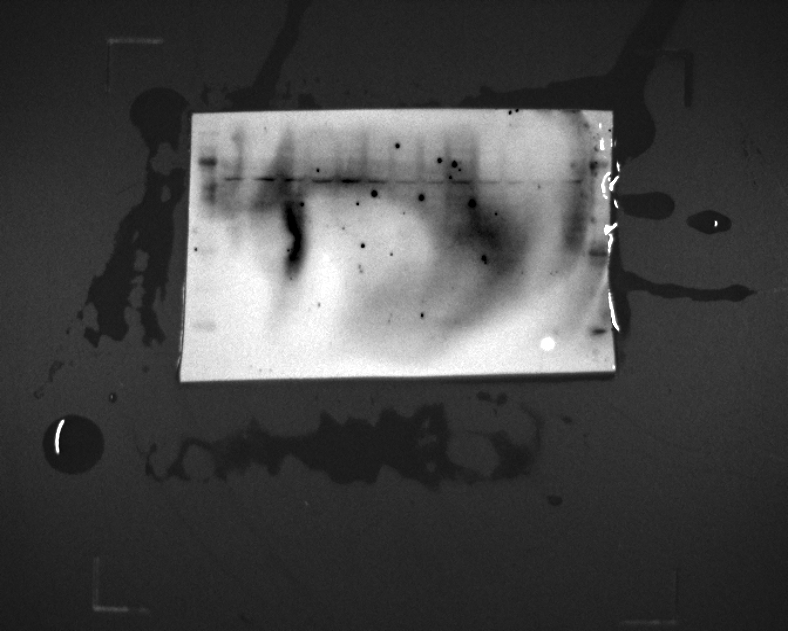


ZNF583


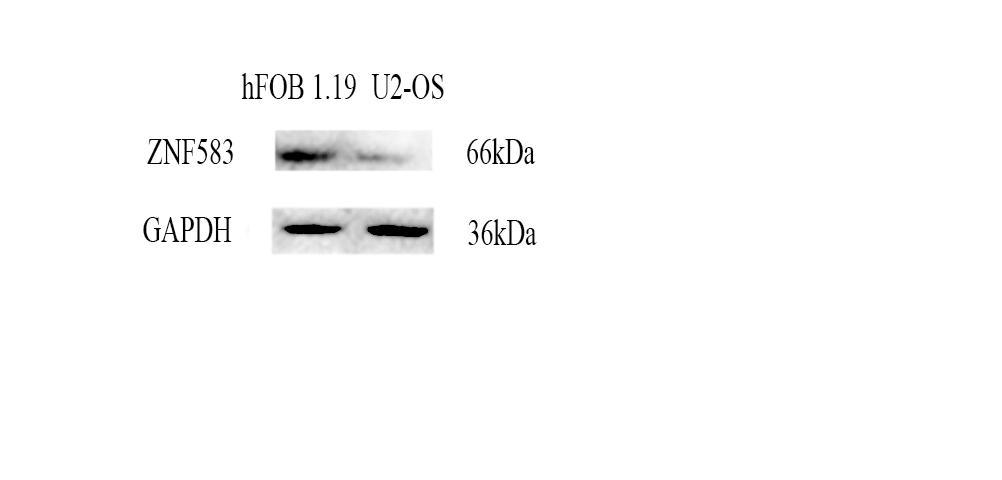


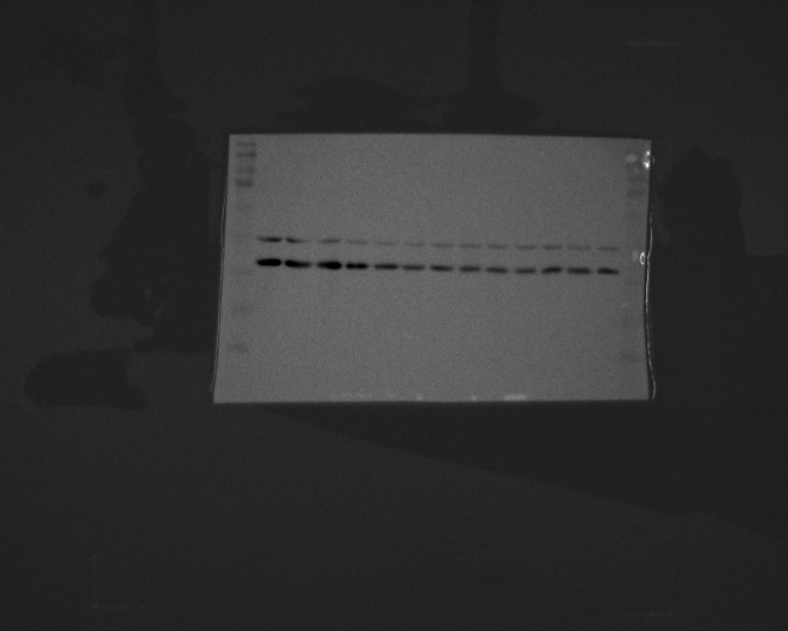


GAPDH

Supplement: Supplementary file 10 — Supplementary Information 10. [file 41598_2023_47367_MOESM10_ESM.docx]
