# Supplementary material for: Single-cell and bulk RNA sequencing reveals Anoikis related genes to guide prognosis and immunotherapy in osteosarcoma
Source: Sci Rep. 2023 Nov 18;13:20203. doi: 10.1038/s41598-023-47367-3 (PMC10657454; doi:10.1038/s41598-023-47367-3)
Supplement: Supplementary file 11 — Supplementary Legends. [file 41598_2023_47367_MOESM11_ESM.docx]

Supplementary Figure 1 | The heatmap shows the expression of the top 8 marker genes in PC

Supplementary Figure 2 | (A) Variations in the activation of biological pathways between osteosarcoma (OS) tumor cells and normal cells. (B) Enrichment scores of specific signaling pathways in non-malignant and malignant cells within each sample of osteosarcoma.

Supplementary Figure 3 | Consensus clustering was performed using Anoikis-related Genes to determine their expression in two Anoikis subgroups. (A-B) Subclusters were identified based on minimal crossover between cluster strata. The consensus matrix heatmap revealed that k = 2 effectively separated samples into two distinct subgroups. (C) Principal component analysis was used to analyze distinct Anoikis subtypes. The resulting scatter plot demonstrated the classification of samples based on Anoikis-related Gene expression. (D) A heatmap was generated to display the expression of Anoikis-related Genes and clinical features in the two subgroups. Low expression is indicated by blue, while high expression is indicated by red. (E) Kaplan-Meier plots were used to compare overall survival between the two subgroups. CDF (cumulative distribution function)

Supplementary Figure 4 | (A) Consistency clustering was used to generate a colored heat map of the consistency matrix for k=3. The gradient color scheme denoted the consistency values between 0 and 1, with white denoting 0 and dark blue denoting 1. (B) Consensus Cumulative Distribution Function (CDF) plot was generated under k = 2–10, where the number of k represents the number of groups after unsupervised clustering. (C) The Kaplan-Meier plotter was used to estimate the survival curves of the geneClusters (P <0.001, Log-rank test). (D) LASSO parameter profiles of the genes in the training cohort were generated. (E) A parameter profile plot with the log(λ) was created. (F) Differences in riskscore among Anoikis subgroups were determined using the Kruskal-Wallis test. (G) Differences in riskscore among geneClusters were revealed by the Kruskal-Wallis test. (H) A heatmap annotated with age, gender, cluster, and genomic phenotypes (geneCluster) was generated. (I) An alluvial diagram of cluster, geneCluster, risk, and survival status (fustat) was created. (*P < 0.05; **P < 0.01; ***P < 0.001; ns, no significance).

Supplementary Figure 5 | Prognostic analysis of the 3-gene signature in the test cohort and validation cohort (A-B) The risk score curve shows the distribution of the model and the median score in the test and validation cohort, respectively. (C-D) Principal component analysis (PCA) plots were generated for the test and validation cohort. (E-F) Survival analysis was conducted in both the test and validation cohort. (G-H) The area under the curve (AUC) was calculated for both the test and validation cohort to verify the prognostic performance of the 3-gene signature model.

Supplementary Figure 6 | (A) Correlation between the expression of ZNF583, CGNL1, and CXCL13 genes and immune cell infiltration (B) Comparative Analysis Across Cancers Highlights Signature's Specificity and Relevance (DDS: Disease-Specific Survival, OS: Overall Survival, PFI: Progression-Free Interval)

Supplementary Figure 7 | Independent prognostic validation of the three-gene signature and its association with chemotherapy: Forest plot of univariate Cox regression analysis and prognostic analysis among risk score and clinicopathological features. (A) Results of univariate analysis. (B) Results of multivariate analysis. The signature with high-risk scores was associated with high sensitivity to chemotherapy drugs such as (C) Cediranib, (D) Crizotinib, (E) Cyclophosphamide, (F) Dactinomycin, (G) Dasatinib, and (H) Entospletinib, whereas the low-risk group was related to (I) Lapatinib, (J) Sapitinib, and (K) Ulixertinib treatment.

Supplementary Figure 8 | Construction and validation of the riskscore-related nomogram. (A) The nomogram was developed to predict the 1-year, 3-year, and 5-year overall survival of OS patients. (B) Calibration plots of the nomogram were generated to evaluate the accuracy of the predictions for 1-, 3-, and 5-year overall survival. (C) Decision curve analysis (DCA) was performed to compare the prognostic performance of the gender, age, risk, and nomogram models for 5-year overall survival. (D) The time-dependent Receiver Operating Characteristic (ROC) curve was plotted to verify the prognostic performance of the nomogram. The Area Under the Curve (AUC) was calculated
